# *ALMS1*-Deficient Fibroblasts Over-Express Extra-Cellular Matrix Components, Display Cell Cycle Delay and Are Resistant to Apoptosis

**DOI:** 10.1371/journal.pone.0019081

**Published:** 2011-04-26

**Authors:** Elisabetta Zulato, Francesca Favaretto, Caterina Veronese, Stefano Campanaro, Jan D. Marshall, Sara Romano, Anna Cabrelle, Gayle B. Collin, Barbara Zavan, Anna S. Belloni, Enrica Rampazzo, Jürgen K. Naggert, Giovanni Abatangelo, Nicola Sicolo, Pietro Maffei, Gabriella Milan, Roberto Vettor

**Affiliations:** 1 Department of Medical and Surgical Sciences, University of Padua, Padua, Italy; 2 Department of Biology, CRIBI (Centro Ricerca Interdipartimentale Biotecnologie Innovative), University of Padua, Padua, Italy; 3 The Jackson Laboratory, Bar Harbor, Maine, United State of America; 4 Department of Clinical and Experimental Medicine, University of Padua, Padua, Italy; 5 Department of Histology, Microbiology and Biomedical Technologies, University of Padua, Padua, Italy; 6 Department of Human Anatomy and Physiology, University of Padua, Padua, Italy; 7 Department of Oncology and Surgical Sciences, University of Padua, Padua, Italy; Institute of Science and Technology Austria, Austria

## Abstract

Alström Syndrome (ALMS) is a rare genetic disorder (483 living cases), characterized by many clinical manifestations, including blindness, obesity, type 2 diabetes and cardiomyopathy. ALMS is caused by mutations in the *ALMS1* gene, encoding for a large protein with implicated roles in ciliary function, cellular quiescence and intracellular transport. Patients with ALMS have extensive fibrosis in nearly all tissues resulting in a progressive organ failure which is often the ultimate cause of death. To focus on the role of *ALMS1* mutations in the generation and maintenance of this pathological fibrosis, we performed gene expression analysis, ultrastructural characterization and functional assays in 4 dermal fibroblast cultures from ALMS patients. Using a genome-wide gene expression analysis we found alterations in genes belonging to specific categories (cell cycle, extracellular matrix (ECM) and fibrosis, cellular architecture/motility and apoptosis). ALMS fibroblasts display cytoskeleton abnormalities and migration impairment, up-regulate the expression and production of collagens and despite the increase in the cell cycle length are more resistant to apoptosis. Therefore *ALMS1*-deficient fibroblasts showed a constitutively activated myofibroblast phenotype even if they do not derive from a fibrotic lesion. Our results support a genetic basis for the fibrosis observed in ALMS and show that both an excessive ECM production and a failure to eliminate myofibroblasts are key mechanisms. Furthermore, our findings suggest new roles for ALMS1 in both intra- and extra-cellular events which are essential not only for the normal cellular function but also for cell-cell and ECM-cell interactions.

## Introduction

Alström Syndrome [ALMS (MIM #203800)] is a rare, autosomal recessive monogenic disease characterized by a wide spectrum of clinical manifestations involving multiple organs, including blindness, severe insulin resistance, type 2 diabetes, obesity and cardiomyopathy that progressively affects multiple organ systems [1].

Alström Syndrome is caused by mutations in *ALMS1*, a large >230 kb gene located on chromosome 2p13 with ubiquitous expression in most tissues affected [2], [3]. The ALMS1 protein localizes to the centrosomes and basal bodies of ciliated cells, and roles in microtubular organization, intracellular transport, and cilia assembly or function have been suggested [4], [5], [6]. RNA interference knockdown experiments indicate that the total lack of ALMS1 impairs cilia formation [7], supporting the inclusion of ALMS in a wide class of human genetic disorders called “ciliopathies” [8].

Pathological observations from post-mortem ALMS specimens reveal extensive fibrosis in most organs [9]. The fibrotic damage observed in ALMS patients, most notably in cardiac, pulmonary, hepatic and renal tissue, appears to strongly influence the patients' prognosis and is often associated with premature death.

Fibrosis is a complex tissue disease [10], [11] in which fibroblasts are the main cell type involved. During tissue repair and fibrosis, fibroblasts shift from a quiescent state regulating ECM turnover to a proliferative and contractile ‘activated’ phenotype (myofibroblast) [12], in which they secrete higher ECM levels and several growth factors and cytokines [11], [13]. Removal of activated fibroblasts seems to be crucial in the resolution of the fibroproliferative responses and an imbalance between fibroblast proliferation and apoptosis could lead to pathological scarring or to a progressive fibrosis [13], [14]. Dermal fibroblasts provide a good model to study fibroblast behavior and mechanisms of pathological regulation during inflammation and wound healing, in particular in fibrotic disorders such as keloid formation [15] and scleroderma [16].

We focused on the role of *ALMS1* mutations in the development of fibrosis using primary cultured fibroblasts of 4 ALMS patients obtained from derma, a region with no signs of fibrotic lesions. To determine whether there were motility/cytoskeleton alterations in ALMS fibroblasts, we characterized morphological features in 2D (bi-dimensional) and 3D (tri-dimensional) cultures by optical and electron microscopy. Using gene expression arrays, we found the modulation of genes related to “cell cycle”, “apoptotic pathways” and to “cellular architecture and motility”. Altogether, our results show that ALMS fibroblasts up-regulate collagen expression and secretion, display a longer cell cycle, and are more resistant to apoptotic stimuli.

## Materials and Methods

### Subjects

Four patients (PT1–PT4) with typical clinical features of ALMS (3 males/1 female, age 24–37 years) participated in this study. PT2 and PT3 are brothers. Genetics and clinical characteristics are reported in Table 1.

**Table 1:** Genetics and clinical characteristics of four ALMS patients studied.

	PT1	PT2	PT3	PT4
	◯	□	▽	◊
*ALMS1* mutation*	c.[8938C>T]	c.[3425C>G]	c.[3425C>G]	c. [2164A>T]
	+[8938C>T]	+[3425C>G]	+[3425C>G]	+[11313_11316delTAGA]
	(exon 10)	(exon 8)	(exon 8)	(exon 8)+(exon16)
	p.Q2980X	p.S1142X	p.S1142X	p.K722X+D3771fsX3790
Age	37 years	36 years	24 years	29 years
Gender	female	male	male	male
BMI (kg/m^2^)	22,2	27,7	27	34,4
Short stature	Yes	Yes	Yes	Yes
Retinopathy	Yes	Yes	Yes	Yes
	(1 year)	(3 months)	(3 months)	(1 month)
Hearing loss	Yes	Yes	Yes	Yes
	(19 years)	(6 years)	(1 year)	(10 years)
Type 2 diabetes	Yes	IGT	No	Yes
	(32 years)	(34 years)		(27 years)
Dyslipidemia	Yes	Yes	Yes	Yes
	(25 years)	(13 years)	(4 months)	(10 years)
Cardiomyopathy	No	No	No	No
Pneumopathy	No	No	No	No
Hepatic disorders**	Mild	Mild	Mild	Yes
	(25 years)	(12 years)	(24 years)	(10 years)
Nephropathy***	Mild	Mild	No	Yes
	(32 years)	(35 years)		(24 years)
Hypogonadism	No	Yes	Yes	Yes

The parameters were measured at the time of dermal biopsies. The age of first diagnosis of disease is reported in parenthesis.

*The nomenclature was established according to den Dunnen *et al.* [29]; the number of the corresponding exon in which the mutation is localized is reported in parenthesis.

**Elevation of transaminases or ultrasographic signs of liver steatosis.

***Elevation of Blood Urea Nitrogen or creatinine or abnormal Glomerular Filtration Rate assessed by kidney scintigraphy.

Three healthy, normal weight control subjects (C1–C3) (2 females/1 male, age 53–74 years) with no endocrine or metabolic alterations served as control subjects. We evaluated some senescence markers (mRNA expression of age-associated genes, telomere length and senescence associated (SA)- β galactosidase activity) and showed that control and ALMS fibroblasts were not influenced by the different age of the donors (Figure S1 and S2.)

### Ethics Statement

Each subject gave informed written consent for dermal biopsy and use of gDNA and cDNA; the Ethical Committee of Padua Hospital approved the research protocol.

### Mutation analysis

cDNA obtained from dermal fibroblasts and gDNA isolated from peripheral blood samples of ALMS patients were amplified using a standard PCR protocol with HotStarTaq Master Mix Kit (QIAGEN GmbH, Hilden, Germany). Primer sequences are available on request. Amplicons were purified, sequenced using ABI PRISM Big Dye Terminator Cycle sequencing Ready Reaction Kits and analyzed by ABI 3100 Sequencing Analyzer (Applied Biosystems, Carlsband, CA, USA). Sequences were compared with the unaffected control and the mRNA reference sequence (NM_015120.4).

### Dermal fibroblast primary cultures

#### Bidimensional (2D) cultures

Dermal biopsy was performed from the glabrous surface of the forearm of ALMS patients and healthy controls. Fibroblasts were maintained in DMEM high glucose (HG) medium (Dulbecco's Modified Eagle Medium GIBCO, Invitrogen LifeTecnologies, Paisley, UK), 150 U/ml streptomycin, 200 U/ml penicillin, 2 mM glutamine, 1 mM HEPES (GIBCO) (standard medium, SM), containing 10% FBS (fetal bovine serum) (GIBCO). All experiments described were performed after cell synchronization achieved by growth to sub-confluency and checked by flow cytometry analysis of the cell cycle. Primary cells were used between passages 3 and 14, and experiments were performed using fibroblast cultures at the same passage.

#### Tridimensional (3D) cultures

Fibroblasts were seeded in scaffolds of HYAFF-11™ (1×1 cm) (supplied by Fidia Advanced Biopolymers, Abano Terme, Italy), at a density of 10^6^ cells/cm^2^, in 10% FBS SM.

### Histological and morphological analysis

Standard hematoxylin and eosin staining was performed in 2D and 3D-fibroblasts fixed in formalin and paraffin-embedded (SIGMA, Sigma Aldrich, St Louis, MO, USA). Cell length was measured using Leica IM1000 Image Manager software (Leica Microsystem, Heerbrugg, Switzerland). Cell number was assessed by cell counting in Bürker chambers, after staining with 0.2% trypan blue. Fibroblasts harvested by trypsinization were analysed on a FACS Calibur flow cytometer (Becton Dickinson Immunocytometry System, San Jose, CA, USA).

The cytochemical staining for (SA)- β galactosidase was performed as previously described [17]. Briefly, cells seeded at the density of 10^4^ cells/cm^2^ were fixed using 3% formaldehyde for 5 minutes. Subsequently, cells were rinsed in PBS and incubated at 37°C for 16 hours with the following staining solution: 1 mg/ml 5-bromo-4-chloro-3-indolyl-β-D-galactopyranoside, (X-gal) (Merck KGaA, Whitehouse Station, NJ, USA), 5 mM potassium ferrocyanide, 5 mM potassium ferricyanide, 150 mM NaCl, 2 mM MgCl2 and 40 mM citric acid pH 6.0 (SIGMA). Representative fields were photographed at 32× magnification using Remote Capture software (Canon) and the DM IL inverted microscope (Leica).

3D-cultures were fixed in 2.5% glutaraldehyde in 0.1 M phosphate buffer pH 7.4 for 3 hours, post-fixed with 1% osmium tetroxide, dehydrated, and embedded in araldite (SIGMA). Ultrathin sections were stained with uranyl acetate and lead citrate, and analyzed with a Philips EM400 electron microscope (Philips, Eindhoven, The Netherlands).

### RNA extraction and DNAse treatment

Total RNA was extracted from 2D cultures with an RNeasy Mini Kit (QIAGEN GmbH, Hilden, Germany). RNA was quantified using NanoDrop technology (Fisher Scientific SAS, Illkirch Cedex, France), quality-checked using an Agilent 2100 Bioanalyzer (Agilent Technologies, PaloAlto, USA) and treated with DNase Treatment & Removal Reagents (Ambion, Inc, Austin, TX, USA).

### DNA microarray experiments

We used slides spotted by the Microcribi Core Facility at the University of Padua (http://microcribi.cribi.unipd.it), containing 21329 oligonucleotides from Operons 70 mer oligo collection (Human Version 2.0) spotted in duplicate (GPL2136136 record in the GEO database) on MICROMAX glass slides SuperChip I (Cat No MPS696, PerkinElmer Life Sciences Inc., Boston, MA, USA).

One DNAse treated total RNA sample (1.5 µg), extracted from each fibroblast cultures of controls and patients (n = 7), was subjected to linear amplification with SuperScript™ Indirect RNA Amplification System (Invitrogen). The resulting aminoallyl labelled aRNA was coupled with fluorescent dyes Cy3 and Cy5 (CyDye Post Labelling Reactive Dyes, GE Healthcare Life Sciences, Piscataway, NJ, USA). Each patient's RNA sample (PT1, PT2, PT3, PT4) was co-hybridized with a control's pooled RNA (C1+C2+C3) and analyzed in replicate with dye swap, for a total of 8 slides (n = 8). 1.5 µg of aRNA labeled with 200 pmoles of fluorophore were used for each hybridization performing as previously described [18] with the ArrayBooster™ (Advalytix, Brunnthal, Germany) at 48°C for 12 h. The fluorescence was detected with the ScanArray Lite Scanner and analyzed using QuantArray Software (PerkinElmer). All data are MIAME compliant and the raw data have been deposited in a MIAME compliant database, ArrayExpress database (http://www.ebi.ac.uk/arrayexpress/), with accession number E-MEXP-2492.

### RT-Real time PCR

Two DNAse treated RNA (2 µg) samples, independently extracted from fibroblast cultures of each subjects (n = 14) and different from the RNA samples used for microarray analysis, were reverse-transcribed for 1 h at 37°C with 150 ng random hexamers, 0.5 mM dNTPs, 20 U of RNAsin Ribonuclease Inhibitor and 200 U of M-MLV (Moloney murine leukemia virus) Reverse Transcriptase (Promega, Madison, WI, USA). Quantitative PCR was performed using fluorigenic SYBR Green PCR Master Mix (Invitrogen) on DNA Engine Opticon™ 2 Continuous Fluorescence Detection System (MJ Research, MA, USA). Oligonucleotide sequences and reaction conditions are reported in the Table S1. Each cDNA sample (5 ng) was assayed in duplicate in at least two independent qPCR reactions (n = 56). Results were normalized with *HMBS* (hydroxymethylbilane synthase) mRNA content and reported as arbitrary units ratio.

### [^3^H]-proline incorporation

2×10^4^ cells were cultured for 24 h in 10% FBS SM, followed by 24 hours in 0.1% FBS SM. 0.5 µCi/well [^3^H]-proline (GE Healthcare Life Sciences) in 0.1% FBS SM was added and cells were cultured for the following 72 h. Cells were lysed with 10% SDS while the culture medium was collected, incubated 30 minutes at 4°C with an equal volume of 20% trichloric acid (TCA) and centrifugated (13000 g for 15 min) for protein precipitation. The pellet was washed with 10% TCA and dissolved in 200 µl 0.3 M NaOH, 0.3% SDS. Radioactivity was quantified using a β-counter (PerkinElmer) and normalized to DNA content, estimated by measuring the OD at 260 nm. Each sample was assessed in duplicate and every experiment was performed at least 3 times.

### Cell cycle length estimation and proliferation assay

Fibroblasts were grown in 10% FBS SM to approximately 80% confluence, and 2.5×10^3^ cells were plated in 96-well microtiter plates in 10% FBS SM or in 2% FBS SM. In a second set of experiments, fibroblasts to approximately 80% confluency were maintained in serum free condition for 48 hours and then plated in 96-well microtiter plates in 10% FBS SM at the same density above reported. At time 0 and after 72 hours cell number was determined using CellTiter-Glo® Luminescent Cell Viability Assay (Promega) by a Victor_3_™ luminometer (PerkinElmer) to calculate the cell cycle length. Each sample was assessed in triplicate and analyzed in 3 independent experiments.

### Cell viability assay

10^4^ cells were plated in 96-well microtiter plates in 10% FBS SM and treated with 100 nM thapsigargin (THAP), 100 nM staurosporine (STP) and 100 ng/ml TNF-α for 48 hours, with 100 µg/ml cycloheximide (CX) and 100 µM C_2_-ceramide (C_2_-C) (SIGMA) for 24 hours. MTT [3-(4,5-dimethylthiazol-2-yl)2,5-diphenyltetrazolium bromide] solution in PBS was added to each well (0.4 mg/ml) for 3 hours at 37°C. Medium was removed and the formazan precipitate was dissolved in dimethylsulfoxide (SIGMA). OD was measured at 550 nm with a reference at 620 nm, using a Victor_3_™ spectrometer (PerkinElmer). Cell viability was calculated as a percentage of absorbance in comparison with untreated cells. Each sample was assessed in triplicate and each experiment was performed at least 3 times.

### Cell cycle analysis

1×10^6^ cells were washed with PBS, fixed with 70% (v/v) cold ethanol for 30 minutes at 4°C, centrifuged (300 g for 8 min) and washed in PBS. Cells were stained with Propidium Iodide (PI) (50 µg/ml), in the presence of RNAse A (0.5 mg/ml) (SIGMA) at 4°C in the dark for 30 minutes. DNA content was analyzed on a FACS Calibur flow cytometer (Becton Dickinson Immunocytometry System).

### Detection of apoptosis by DNA end-labeling (TUNEL)

DNA fragmentation was measured using the DeadEnd™ Fluorimentric TUNEL System (Promega). Fibroblasts were plated on coverslips at 2×10^4^ cells/cm^2^, and upon treatment fixed in 4% paraformaldehyde solution in PBS and permeabilized in 0.2% Triton X-100 in PBS at r.t. for 5 minutes. DNA was labeled with fluorescein-12-dUTP by using terminal deoxynucleotidyl transferase enzyme (rTdT) for 1 hour at 37°C in humidified chamber. The reaction was terminated in 300 mM sodium chloride, 30 mM sodium citrate buffer at r.t. for 15 minutes. Nuclei were counter-stained with 4,6-diamidino-2-phenylindole (DAPI, Vectashield®, Vector Laboratories, Burlingame, CA, USA). Coverslips were observed under a Leica fluorescence microscope (Leica DM LB2, Leica Microsystem). Alternatively, fibroblasts upon treatment were harvested with 25% trypsin (v/v) and 2×10^6^ cells were fixed in 1% paraformaldehyde solution in PBS for 20 minutes at 4°C and permeabilized in 70% (v/v) cold ethanol at −20°C for 4 hours. DNA was labeled as previously described and the labeling reaction was terminated by the addition of 20 mM EDTA. Cells were stained in the dark with PI (50 µg/ml), in the presence of RNAse A (0.5 mg/ml) (SIGMA) at 4°C for 30 minutes, and analyzed on a FACS Calibur flow cytometer (Becton Dickinson Immunocytometry System).

### Telomere length measurement by quantitative Real time PCR

DNA was extracted by the standard phenol/chloroform method from fibroblasts cells. Telomere length was quantified as previously described [19].

### Statistics and microarray data analysis

Results are reported as mean value ± standard error (SEM). Statistical comparison between two sets of data was performed using unpaired Student's *t* test (two-tailed) or unpaired Mann Whitney test (two-tailed) in respect to the distribution and size of samples. Differences were considered significant at *P*<0.05.

Low intensity spots of microarray were removed from both channels applying Z-score transformation and we balanced raw intensities applying LOWESS (locally weighted scatter plot smoothing), discarding outlier values from the analysis for every slide. For each slide, we removed incongruent replicates calculating the ratio between ch1 and ch2 intensity for the 2 replicate with dye swap (respectively R1 and R2) and the standard deviation (SD). After that, we calculated the log2(R1/R2) and excluded the spots showing the value higher than 3SD. We considered only the genes that have the same expression pattern in all patients analyzed. Subsequently, we calculated the geometric means for the filtered genes and applied one class SAM (Significance Analysis of Microarrays) analysis to identify the differentially expressed genes by grading each gene using a modified *t* test [20]. For each gene SAM software calculates a false discovery rate (FDR) which is an estimation of the percentage of genes identified as differentially expressed by chance. In our analysis only the genes with 0% FDR were included in the subsequent analysis. To analyze the biological meaning of the selected genes we used GoMiner software (Gene Ontology, http://www.geneontology.org/) and the KEGG database (Kyoto Encyclopedia of Gene and Genome, http://www.genome.jp/kegg/). To avoid the loss of information regarding differentially expressed genes we performed also a careful analysis of published literature and we will refer to this as “gene by gene” approach.

## Results

### Size, shape and ultrastructural evaluation of ALMS fibroblasts

**Figure 1:**
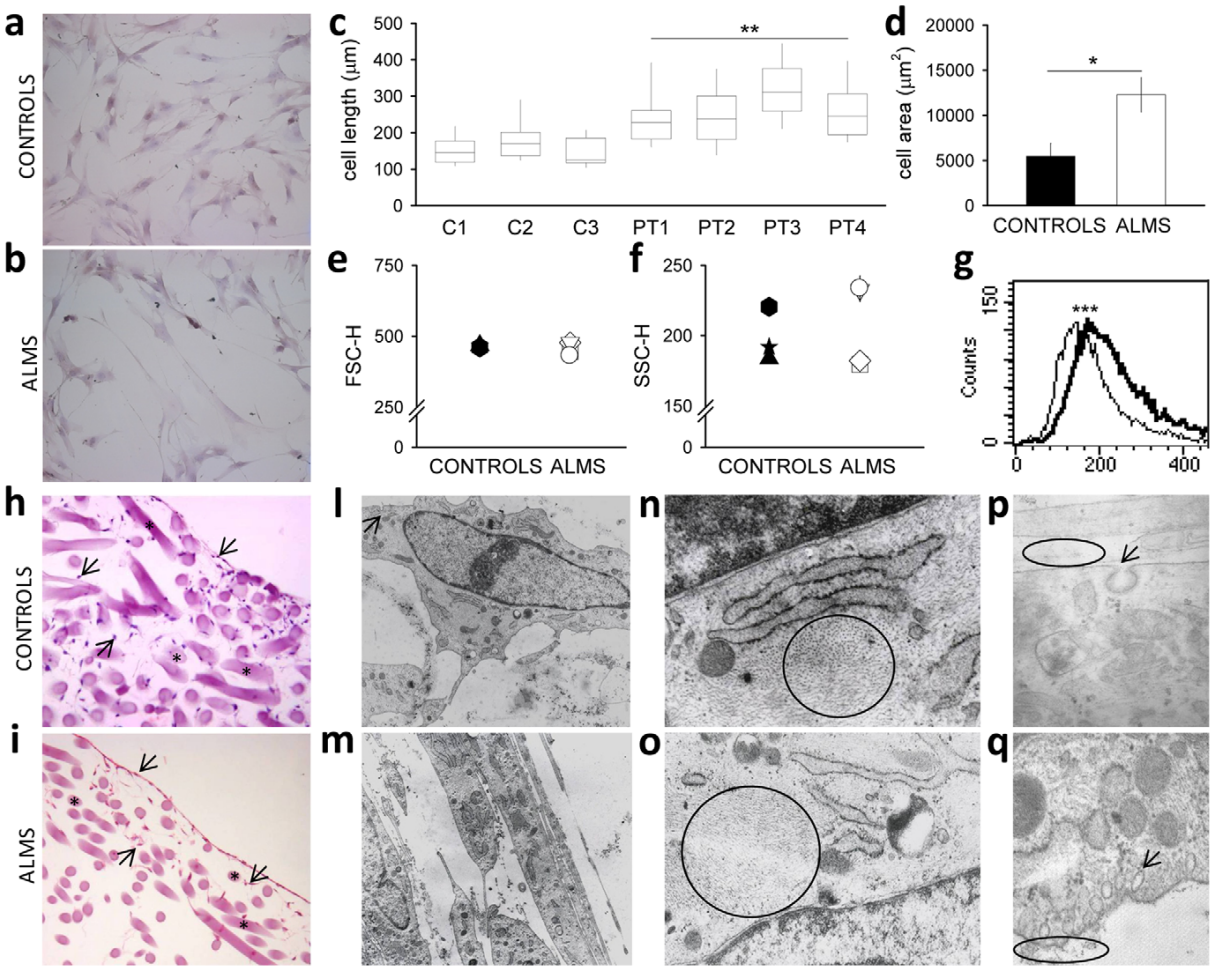
Size, shape and ultrastructural evaluation of ALMS fibroblasts. (a) Fibroblasts of healthy control (C3) and (b) ALMS patient (PT3) were grown on standard tissue culture (2D cultures) coverslips and stained with hematoxylin-eosin (magnification 20×). (c) Cell length of about 40 fibroblasts per subject was quantified by measuring the longitudinal cell length and was plotted as mean (horizontal line), maximal and minimal length values (vertical line). **P<0.01 ALMS fibroblast length mean *versus* controls length mean. (d) The area covered by fibroblast cells during exponential growth was estimated by cell counting in Bürker chambers, after 0.2% trypan blue staining. Results are reported as mean ± SEM. *P<0.05. ALMS fibroblasts were compared with controls by flow cytometric analysis: (e) the resulting forward (FSC) and (f) side light scatter (SSC) mean intensity values are shown for each subject. Controls: C1, black triangle; C2, black star; C3, black diamond; Patients: PT1, white circle; PT2, white square; PT3, white triangle; PT4, white diamond (see also Table 1). Panel g reports the SSC distribution of fibroblasts from C1 (black line) and PT1 (grey line) showing the highly significant shift (***P<0.001, as determined by the Kolmogorov-Smirnov analysis according to the Macintosh CELLQuest software user's guide, Becton Dickinson). (h) Fibroblasts of healthy control (C2) and (i) ALMS patient (PT2) were cultured in HYAFF-11™ scaffolds (3D-cultures) and stained with hematoxylin-eosin (magnification 5×; * biomaterial scaffolds; → fibroblast cells). Transmission electron microscopy was performed in healthy control (C2) (l–n–p) and ALMS patient (PT2) (m–o–q) on 3D-cultured cells. Control fibroblasts showed a normal phenotype, with typical bipolar morphology (l), cytoplasm rich in perpendicular oriented microfilaments (n, ring), the presence of normal pinocytic vesicles (l arrow and p) and probably collagen fibers in the extracellular space (p, ring). In contrast, ALMS fibroblasts appeared as elongated cells tightly adherent to each other and showed well-defined long cytoplasmic extensions (m). Microfilaments (o, ring) were arranged in a unique direction, parallel to the long axis of the cells. A large amount of exocytic vesicles and probably collagen fibrils (q, arrow and ring, respectively) suggest active secretion. Magnification: l–m = 5000×; n–o = 25000×; p = 40000×; q = 30000× (see also Figure S3 and S4).

ALMS cells (PT1–PT4) are more elongated, with well defined cytoplasmic extensions, compared to controls (C1–C3) (Figure 1a,b,c) and occupied a larger surface than healthy cells (Figure 1d). Nevertheless in suspension ALMS and control fibroblasts displayed similar dimensions (Figure 1e), whereas two of the mutated ones possessed a major cellular complexity (Figure 1f,g and Figure S3).

3D cultures of control fibroblasts showed normal growth inside the scaffolds (Figure 1h), whereas ALMS fibroblasts did not penetrate into the biomaterial, with unaffected vitality, giving rise to a pluri-stratification of cells in the upper side of the biomaterials (Figure 1i and Figure S4).

Transmission electron microscopy (TEM) analysis confirmed the normal morphology of control fibroblasts, appearing typically bipolar, occasionally tripolar (Figure 1l) with an ovoid nucleus and two to five nucleoli. The cytoplasm presented both rough and smooth reticulum, numerous mitochondria and occasional lysosomes. The submembranous cytoplasm was also rich in microfilaments found in perpendicular arrangement with the nuclei (Figure 1n). Normal secretion was indicated by the presence of pinocytic vesicles in the plasma membrane (Figure 1p). In contrast, ALMS fibroblasts showed elongated nuclei and the presence of well defined cytoplasmic extensions, strongly anchored in the extracellular area between adjacent cells (Figure 1m). The cytoplasm of ALMS fibroblasts was enriched with numerous mitochondria and lysosomes, and contained microfilaments arranged in a unique direction parallel to the nuclei (Figure 1o). An active secretion was suggested by the presence of a large number of exocytic vesicles (Figure 1q and Figure S4).

### Differentially expressed genes in ALMS fibroblasts

**Figure 2:**
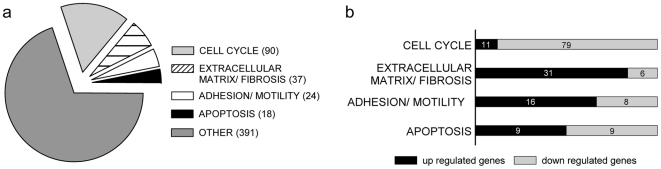
Distribution of differentially expressed genes in ALMS fibroblasts. Fibroblast transcriptional profiles were analyzed by whole genome expression experiments using RNA isolated from healthy controls and ALMS fibroblasts. (a) Pie chart shows the percentage of differentially expressed genes (n = 560) in ALMS fibroblasts clustered in main categories: cell cycle (n = 90), ECM components/fibrosis regulation (n = 37), cellular adhesion/motility (n = 24) and apoptosis (n = 18). (b) Bar chart displays the distribution of up- (black) and down- (grey) regulated genes for each identified group with the number of corresponding genes. A detailed description of the whole genome expression analysis is given in Table S2.

We performed whole genome expression analysis comparing cultured fibroblasts from ALMS patients and healthy controls. Of about 21500 genes, 560 were scored as differentially expressed, 188 of which were up-regulated whereas 372 were down-regulated in ALMS patients. We were able to classify differentially expressed genes in ALMS fibroblasts into 4 groups: cell cycle, extracellular matrix components/fibrosis regulation, cellular adhesion/motility and apoptosis (Figure 2, Table 2 and Table S2).

**Table 2:** Gene Ontology distribution of differentially expressed genes in microarray analysis.

BIOLOGICAL PROCESS	GO ID	Changed/Total genes	P-Value Changed genes	CELLULAR COMPONENT	GO ID	Changed/Total genes	P-Value Changed genes	MOLECULAR FUNCTION	GO ID	Changed/Total genes	P-Value Changed genes
**Cell cycle**	7049	53/539	0	**Collagen**	5581	9/30	0	**Growth factor binding**	5520	10/19	0
	75	9/35	0		5583	6/10	0		19838	12/50	0
	87	24/116	0		5588	2/3	0.0072	**Binding to extracellular matrix constituent**	5540	4/11	0.0015
	278	28/157	0		5598	1/1	0.0498		5518	4/14	0.0041
	279	27/147	0		5595	1/1	0.0498		5521	9/72	0.0072
	7067	24/114	0		5591	1/1	0.0498		5539	9/72	0.009
	51301	18/126	0		5586	1/1	0.0498		30247	9/73	0.0099
	7088	8/33	0.0002		5582	1/1	0.0498		1871	9/76	0.0127
	7093	5/12	0.0002	**Microfibril**	1527	3/3	0.0001	**Extracellular matrix constituent**	5201	10/69	0.002
	74	32/370	0.0015	**Fibril**	43205	3/4	0.0005	**DNA dependent ATPase activity**	8094	6/28	0.0022
	51726	32/370	0.0015	**Extracellular matrix**	5578	26/308	0.0057	**DNA primase activity**	9008	2/6	0.0324
	51325	8/54	0.0049		31012	26/309	0.0059	**DNA-methyltransferase activity**	3886	2/5	0.0223
	51329	8/54	0.0049	**DNA polymerase complex**	5660	4/4	0	**ss DNA binding**	3697	4/25	0.0333
	82	4/19	0.0129		5659	4/5	0	**DNA polymerase activity**	3887	4/27	0.0428
	7094	2/4	0.0139		5658	3/3	0.0001		30337	1/1	0.0498
	16043	62/975	0.0238		42575	4/7	0.0002	**RR activity**	4748	2/4	0.0139
	8283	32/449	0.0248		5662	2/3	0.0072	**Nucleotidyltransferase activity**	16779	8/80	0.0447
	7346	3/16	0.0422		5663	2/4	0.0139	**Adenosinedeaminase activity**	4000	2/4	0.0139
	7089	2/7	0.0439	**Replisome**	30894	9/12	0	**Microtubule motor activity**	3777	5/30	0.0152
	7064	1/1	0.0498		5657	9/14	0	**Motor activity**	3774	8/78	0.0393
	7095	1/1	0.0498		43601	9/12	0				
**Regulation of cell growth**	1558	14/87	0.0001		43596	9/12	0				
	40008	14/93	0.0002	**RR complex**	5971	1/1	0.0498				
	16049	15/110	0.0003	**Nuclear chromosome**	228	13/50	0				
**Regulation of cdk activity**	79	7/32	0.0008		5694	27/157	0				
**Spindle organization and biogenesis**	7051	9/17	0		794	4/15	0.0053				
	7052	5/11	0.0001		793	4/20	0.0155				
	31577	2/4	0.0139	**Chromosome region**	775	7/22	0.0001				
	212	1/1	0.0498	**Cohesin core heterodimer**	8280	1/1	0.0498				
**DNA replication**	6260	27/131	0	**Kinetochore**	776	6/15	0				
	7049	53/539	0	**Spindle**	5819	7/24	0.0001				
	6270	5/16	0.0008		5816	2/3	0.0072				
	6269	2/3	0.0072	**Microtubule cytoskeleton**	15630	14/140	0.0097				
	6273	2/3	0.0072	**Polar microtubule**	5827	1/1	0.0498				
	6275	3/10	0.0113	**Contractile ring**	5826	1/1	0.0498				
	8156	2/4	0.0139	**Intermediate filament**	45111	8/61	0.0102				
**DNA metabolism**	6259	47/392	0	**Cytoskeleton**	5882	8/61	0.0102				
	51052	6/23	0.0007	**Kinesin complex**	5871	3/11	0.015				
	51053	3/5	0.0011	**Nucleus translation initiation factor 2B complex**	5634	127/2202	0.0285				
**Deoxyribonucleotide biosynthesis and metabolism**	9221	2/2	0.0025		5635	7/66	0.0447				
	9263	2/3	0.0072		5851	1/1	0.0498				
	9186	2/4	0.0139								
	9219	2/4	0.0139								
	9132	2/6	0.0324								
	6221	3/16	0.0422								
	9124	3/17	0.0495								
	9123	3/17	0.0495								
	6231	1/1	0.0498								
	6233	1/1	0.0498								
	6235	1/1	0.0498								
	9139	1/1	0.0498								
**Microtubule cytoskeleton organization and biogenesis**	226	9/36	0								
	7017	13/77	0.0001								
	7018	5/38	0.0387								
	72	1/1	0.0498								

We grouped the identified terms with the corresponding accession numbers (GO ID) in the 3 main Ontologies (Biological Process, Cellular Component and Molecular Function). For each GO ID we reported the number of changed genes in our experiments out of the total genes represented for that class in the array. For each term we reported also the statistic significance values derived from GoMiner analysis (p<0.05).

We observed a down regulation of genes coding for proteins directly involved in cell cycle progression (several cyclins and cyclin dependent kinases), in replication, in centrosome-kinetocore assembly and function, and of genes involved in DNA duplication as those required in the minichromosome maintenance complex (MCM) and in the assembly of the replication complex (*RFC3-5*).

Microarray data showed a strong up-regulation of genes coding for extracellular matrix components and their regulators in ALMS fibroblasts, as several collagen types and proteins involved in the collagen chain assembly [*P4HA1* (prolyl 4-hydroxylase, alpha polypeptide I), *LEPRE1* (leucine proline-enriched proteoglycan1) and *SERPINH1* (serpin peptidase inhibitor, clade H, member 1)].

We also found an up-regulation of genes involved in focal adhesion structures [like *TNS1* (tensin1) and *LIMS2* (LIM and senescent cell antigen-like domains2)] and the dystrophin-glycoprotein complex [*SGCD* (sarcoglycan, delta)], which are usually implicated in the regulation of cytoskeleton-ECM interaction.

We found an increase of mRNA for genes which are directly associated with fibrosis such as *POSTN* (periostin, osteoblast specific factor), *IGFBP3* and *IGFBP5* (insulin-like growth factor binding protein 3 and 5) and the modulation of 4 members of the CCN family [enhanced expression of *CTGF* (connective tissue growth factor), *WISP1* (*WNT1* inducible signaling pathway protein 1) and *CYR61* (cysteine-rich, angiogenic inducer, 61) associated with a down-regulation of *NOV* (nephroblastoma overexpressed gene), recently proposed as inhibitor of the fibrotic process]. Finally, we identified the up-regulation of *ACTA2* (actin, alpha2, smooth muscle, aorta) also known as α*-SMA* (alpha smooth muscle actin), a well established marker for myofibroblast conversion. Although GoMiner software identified only 3 apoptosis related genes [*IL24* (interleukin 24), *GULP1* (GULP, engulfment adaptor PTB domain containing 1), *NALP1* (NLR family, pyrin domain containing 1)], the “gene by gene” approach highlighted the modulation of other 15 transcripts [for example *POST*, *EDIL3* (EGF-like repeats and discoidin I-like domains 3) and *MEF2C* (myocyte enhancer factor 2C)] engaged in the control of stress-induced cell death.

### Increased mRNA expression and deposition of ECM components in ALMS fibroblasts

**Figure 3:**
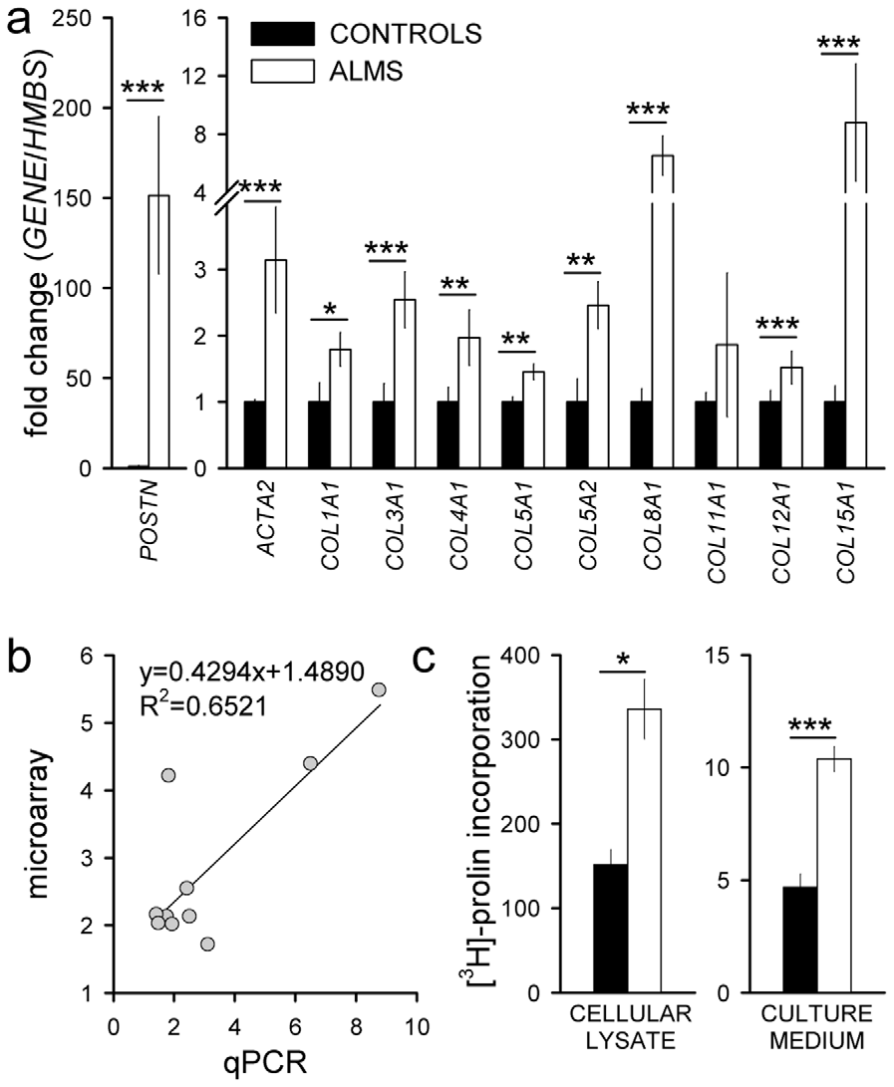
Increased mRNA expression and deposition of ECM components in ALMS fibroblasts. (a) *POSTN*, *ACTA2*, *COL1A1*, *COL3A1*, *COL4A1*, *COL5A1*, *COL5A2*, *COL8A1*, *COL11A1*, *COL12A1*, *COL15A1* transcripts were quantified by qPCR and normalized to *HMBS* mRNA content in control (CONTROLS) and ALMS fibroblasts (ALMS). Results are reported as mean values ± SEM and are expressed as fold change with respect to controls, arbitrarily set as 1 for each of analyzed transcript. *P<0.05, ** *P*<0.01, *** *P*<0.001 ALMS fibroblasts *versus* controls (see also Figure S6). (b) The fold change observed between ALMS and control fibroblasts in microarray analysis was plotted against the fold change measured by qPCR for *ACTA2* and *COLs* transcript. A strong correlation between the two methods was observed. Pearson coefficient = 0.8. (c) Fibroblast collagen protein synthesis and release were determined by [^3^H]-proline incorporation, and expressed as counts per minute normalized to DNA content. *P<0.05, ***P<0.001 ALMS fibroblasts *versus* controls. Black bars correspond to control and white bars to ALMS fibroblasts.

We quantified several fibrosis-related genes in an independent set of cultured fibroblasts by qRT-PCR (Figure S5). As shown in Figure 3a, the expression levels of all analyzed genes (*POSTN*, *ACTA2*, collagens) were higher in ALMS than in control fibroblasts (from 1.5 to 151 fold increase) with a highly significant correlation between microarray data and qPCR determinations (Figure 3b) (Pearson coefficient = 0.8) (see also Figure S6). Collagen production showed a significant 2–fold increase in ALMS fibroblasts compared to controls, as assessed by measuring the [^3^H]-proline incorporation into proteins of cellular lysate (production) and of culture medium (secretion) (Figure 3c). Moreover TGF-β treatment led to a significant induction type I collagen (*COL1A1*) mRNA gene expression, more pronounced in ALMS fibroblasts than in controls (Figure S7).

### ALMS fibroblasts had a longer cell cycle than controls

**Figure 4:**
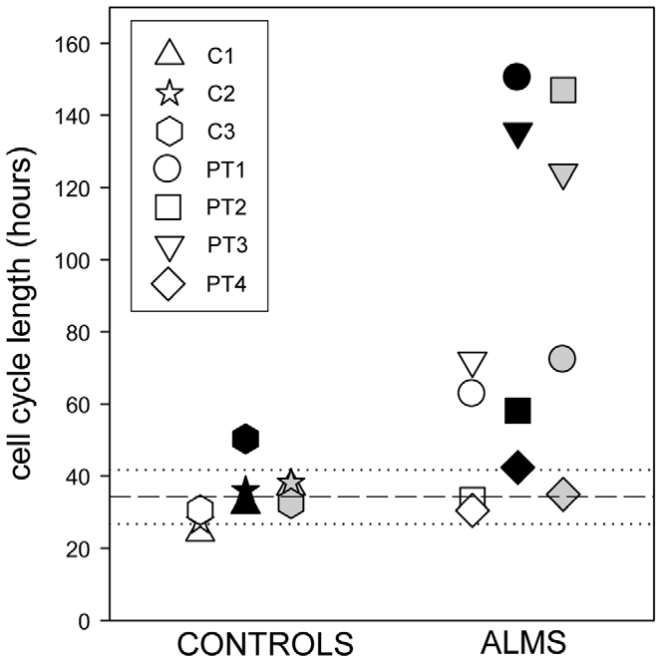
ALMS fibroblasts display a longer cell cycle than controls. Cell cycle length of control (C1–C3) and ALMS fibroblasts (PT1–PT4) was estimated by culture experiments in 10% FBS SM (white symbols), in 2% FBS SM (black symbols), and upon serum deprivation for 48 hour, followed by culture in 10% FBS SM (grey symbols) by comparing the number of viable cells at 0 hours and at 72 hours of culture, using CellTiter-Glo® Luminescent Cell Viability Assay (Promega). The short dash line indicates the mean value of cell cycle length of controls in all growth conditions analyzed, while the dotted lines represent the mean value ± one SD.

We analyzed the cell cycle length of fibroblasts grown under different culture conditions, during the exponential phase of cell proliferation (Figure 4). Three out of four ALMS fibroblasts proliferated at a slower rate in comparison with control fibroblasts in the growth conditions analyzed: normal serum (10% serum), low serum (2% serum) and serum deprivation (48 hours of serum deprivation followed by 10% serum).

Three out of four ALMS fibroblasts were more susceptible to a reduction in cell proliferation rate in response to different culture conditions (low serum or normal serum upon serum starvation), resulting in far slower cell proliferation than controls.

### ALMS fibroblasts were resistant to cell death induced by apoptotic stimuli

**Figure 5:**
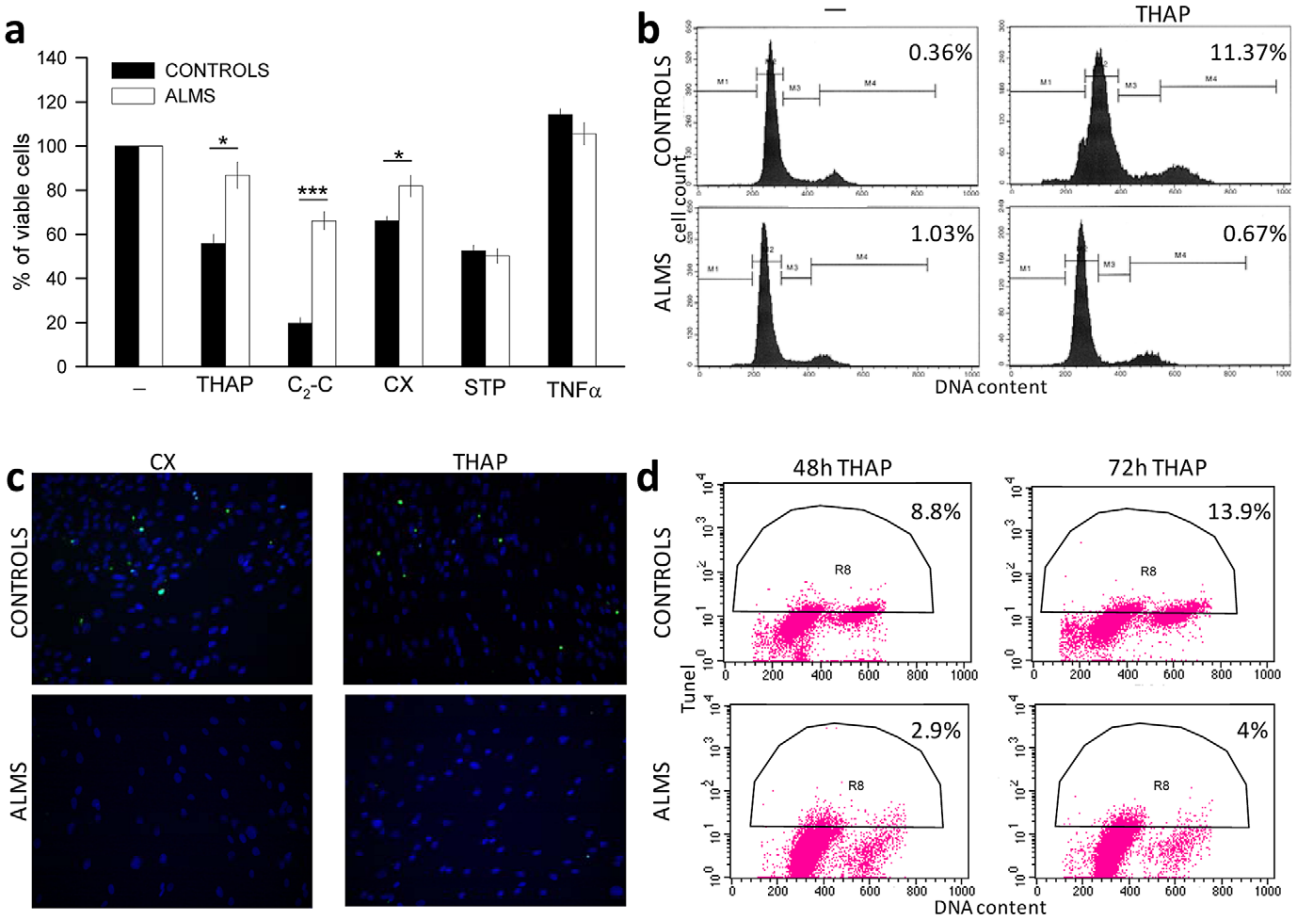
ALMS fibroblasts are resistant to cell death induced by apoptotic stimuli. (a) Fibroblasts of healthy controls and ALMS patients were stimulated with thapsigargin (THAP, 100 nM for 48 hours), C_2_-ceramide (C_2_-C, 100 µM for 24 hours), cycloheximide (CX, 100 µg/ml for 24 hours), staurosporine (STP, 100 nM for 48 hours) and TNF-α (100 ng/ml for 48 hours) in 10% FBS SM. The % of viable cells was determined by MTT assay and shown as mean values ± SEM with respect to unstimulated cells (−) indicated as 100%. *P<0.05, ***P<0.001 cell viability of ALMS fibroblasts *versus* controls. (b) Control (C1) and ALMS fibroblast (PT4) were treated with THAP (100 nM for 48 hours), stained with PI and analyzed by flow cytometry. Results are presented as histograms of cell cycle phase distribution and the reported % represents the increase in sub-G_1_/G_0_ population (M1 region). (c) Control (C1) and ALMS fibroblast (PT1) were grown on glass coverslips and stimulated with CX (100 µg/ml for 24 hours) and THAP (100 nM for 48 hours). Cells were fixed and stained for fluorescence *in situ* DNA end-labelling (TUNEL) (green stain); nuclei were counter-stained with DAPI (blue stain) (magnification = 20×). (d) Control (C1) and ALMS fibroblast (PT1) were treated with THAP (100 nM for 48 and 72 hours). Cells were labelled by TUNEL and PI and analyzed by flow cytometry. Each cytogram shows the TUNEL positivity (ordinate) with respect to DNA content (abscissa), upon 48 and 72 hours of THAP-treatment, respectively. A representative control and patient are reported in panel b, c and d; see also Figure S9 and S10.

Figure 5a showed that the % of viable cells in ALMS fibroblasts was higher than in controls when apoptosis was stimulated with thapsigargin (THAP), C_2_-ceramide (C_2_-C) and cycloheximide (CX). Staurosporine (STP) treatment, however, led to an equal cell death in both patient and control fibroblasts, whereas the survival was not influenced by TNF-α (tumor necrosis factor alpha) stimulation (see also Figure S8).

To determine whether cell death occurred by apoptosis, we measured DNA content by FACS (fluorescence activated cell sorting) analysis upon propidium iodide (PI) staining in two control and two ALMS fibroblast cultures. The sub-G_1_/G_0_ population, which represents the cells with hypodiploid DNA content in the histogram (M1 region), increased to 11.4% in control fibroblasts, whereas it did not change in ALMS fibroblasts after THAP treatment (Figure 5b and Figure S9). ALMS fibroblasts did not display TUNEL-positive nuclei upon CX and THAP treatment unlike controls (Figure 5c and Figure S10). These results were confirmed and quantified by FACS analysis of apoptotic nuclei of two control and two ALMS fibroblasts showed in Figure 5d and Figure S9.

## Discussion

Severe systemic fibrosis is an universal observation in postmortem and biopsy specimens of ALMS patients and may result in early organ failure and death [9]. Whether the fibrotic damage is a direct result of mutations in the *ALMS1* gene or, alternatively, a secondary response to cellular insult due to the loss of ALMS1 function is unknown. However, while hepatic and renal fibrosis could possibly be explained by the metabolic alterations present in ALMS patients, such as obesity and type 2 diabetes, it is difficult to identify a trigger insult causing fibrosis in heart, lung, bladder, testis and ovary.

Fibrosis is often considered a fibroproliferative disorder in which the uncontrolled proliferation of activated fibroblasts and the excessive production of ECM resulted in a functional impairment of affected organs [11].

We did not observe an enhanced proliferative capacity in ALMS fibroblasts. On the contrary, *ALMS1* mutations in the majority of our patients are associated with an increase in cell cycle length and in all patients with a down-regulation of genes directly involved in cell cycle progression, replication and centrosome-kinetocore assembly. Fibroblasts obtained from PT4 displayed a normal cell cycle length and it is noteworthy that only PT4 carried a mutation in exon 16 of *ALMS1*, thus he could preserve some ALMS function in hemizygosity. Additional studies regarding the different disease-causing variants will help us to gain a better understanding of the phenotypic variability observed among ALMS patients.

The localization of ALMS1 to centrosomes and ciliary basal bodies [4], [6] suggests a role in the regulation of cell cycle progression. Primary cilia are regarded as postmitotic structures of quiescent cells [21] and recently were proposed to be involved in the control of cellular growth. ALMS overlaps clinical phenotypes of other ciliopathies, as Barbet-Biedl Syndrome (BBS), and a role for BBS proteins in microtubule organization and cell cycle progression has been hypothesized [22]. The increased doubling time we observed in the majority of ALMS fibroblasts is the first evidence which links ALMS1 function to the regulation of cell cycle progression.

We showed the up-regulation of several genes coding for ECM components and regulators associated with increased collagen synthesis in ALMS fibroblasts. The enhanced intracellular complexity and the active exocytic dynamic assayed by our morphological evaluations similarly suggest a constitutive activated phenotype for dermal ALMS fibroblasts. Furthermore, control fibroblasts became able to express the same *COL1A1* levels as ALMS fibroblasts only upon 48 hour-treatment with TGF-β. Additionally, ALMS fibroblasts remain responsive to TGF-β and the stimulation further increased their *COL1A1* expression more efficiently than controls.

Array experiments showed the mRNA modulation in ALMS fibroblasts of four out of the six matricellular growth factors belongs to the CCN family: *CTGF*, *WISP1*, *CYR61*, *NOV*, all of which function as adaptor molecules connecting the cell surface to the ECM. Constitutive over-expression of *CTGF* has been linked with systemic sclerosis, keloids and other fibrotic skin disorders [23] and the up-regulation of *WISP1*, a WNT signaling pathway target, has been associated with human idiopathic pulmonary fibrosis [24]. We also observed a marked up-regulation of *POSTN*, a protein involved in proper ECM synthesis [25] and in the hypertrophic response following myocardial infarction [26]. The strong periostin overexpression we observed together with the risk described for developing cardiomyopathy in ALMS patients [9] is particularly suggestive of a role of periostin in cardiac remodeling in ALMS.

Taken together all these observations show that ECM deposition is a dominant pathological mechanism of ALMS fibrosis, is constitutively present in mutated fibroblasts, and could be enhanced by the presence of a profibrotic environment.

The 3D-culture experiments showed that ALMS fibroblasts display an inability to migrate into the inner spaces of the scaffold, appearing strongly anchored to adjacent cells. This result is in agreement with expression data indicating the up-regulation of genes involved in focal adhesion structures and suggesting a closer cell-matrix association. Furthermore, our morphological analysis showed the presence of cytoskeletal structures preferentially lined up to the nuclear polarity in ALMS fibroblasts compared to controls. The migration and the orientation of fibroblasts with respect to the collagen fibers are both essential steps in normal tissue repair [13], [27]. Thus, the lack of ALMS1 function could alter the cell polarity and the efficient migration contributing to the generation of fibrosis.

Resistance to apoptosis has been described in fibroblasts isolated from fibrotic lesions in patients with pulmonary fibrosis [28] or with keloids [15]. Our microarray analysis suggests that *ALMS1*-mutated fibroblasts could possess some alterations in genes involved in the apoptotic pathway regulation and partially in the control of stress-induced cell death. Moreover the treatment with some apoptotic inductors (THAP, C_2_-C and CX) is more efficient in controls than in ALMS fibroblasts, indicating that a specific apoptotic pathway could be altered in mutated cells. These results suggest that ALMS fibroblasts display a “prosurvival phenotype” that could play a role in the generation of fibrosis interfering with the control of the fibroproliferative response.

**Figure 6:**
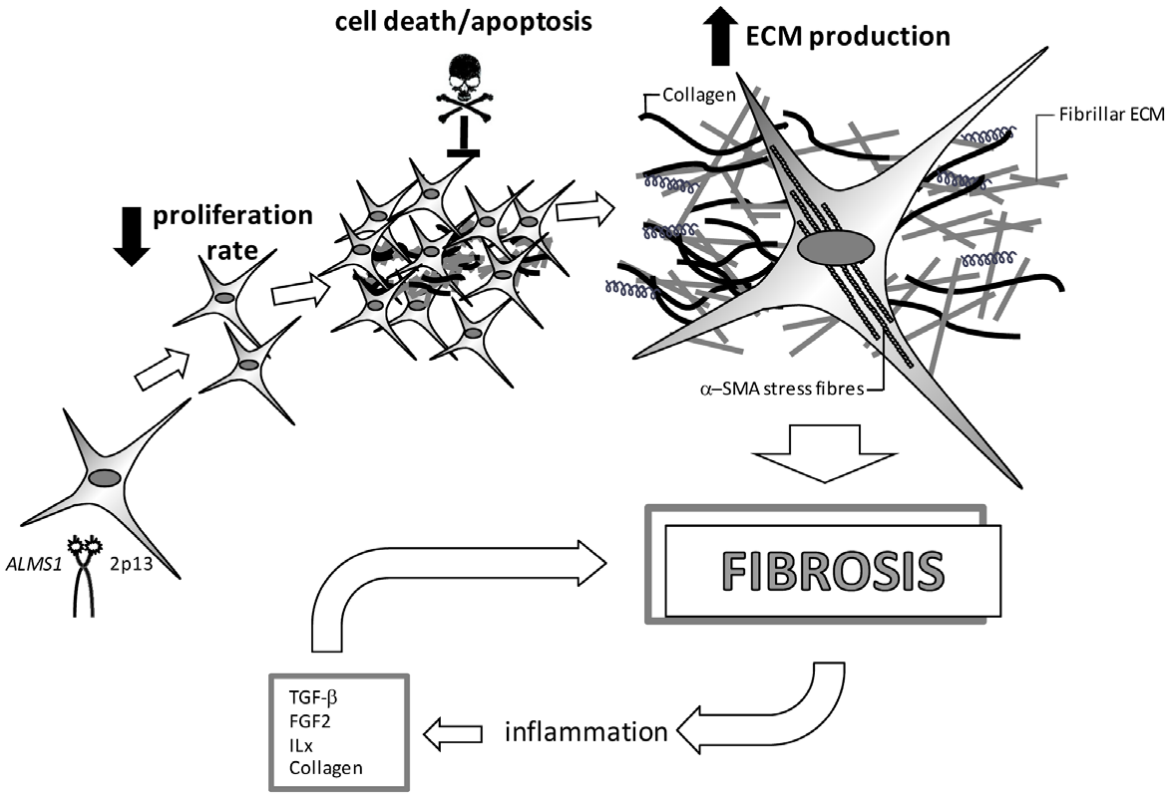
Model of fibrosis in Alström Syndrome. Fibroblasts carrying *ALMS1* mutations display an elongated shape, proliferate slowly, but are still responsive to pro-fibrotic factors and resistant to cell-death stimuli, suggesting that their proliferation is not controlled by apoptosis. *ALMS1* mutated fibroblasts persist, continue to proliferate and to synthesize and secrete high levels of ECM, responsible for progressively remodeling and destroying normal tissue architecture, resulting in fibrosis. The microenvironment could be characterized by an excess of mediators enhancing a cellular pro-fibrotic phenotype, that together with inflammatory reactions could stimulate the fibrosis in an autocrine loop.

Dermal ALMS fibroblasts display a constitutive activation of an α-SMA-myofibroblast like phenotype, indicating autonomous, signal-independent alterations, directly due to mutations in *ALMS1* gene. They divide more slowly and are less susceptible to apoptosis, thus continue to proliferate in an uncontrolled manner and produce an excess of ECM, resulting in fibrosis. Moreover ALMS fibroblasts keep themselves responsive to profibrotic inductors contributing to the perpetuation of fibrosis (Figure 6).

In conclusion, we show that fibrosis in ALMS is a primary defect due to *ALMS1* mutations leading to the fibrotic phenotype described in ALMS patients. Moreover, ALMS1 is a multifunctional protein, with roles in cell cycle progression, migration, apoptosis and ECM production. Further studies investigating the involvement of ALMS1 in both intra- and extra-cellular events will be helpful in unraveling the pathophysiology of ALMS to facilitate the identification of key therapeutic targets.

## Supporting Information

Figure S1 **Age-associated genes expression and relative telomere lengths in control and ALMS fibroblasts.** (a) *p66^Shc^*/*SHC1* p66 isoform, *SIRT1*, *SIRT5*, *c-Myc*/*MYC* and *p21*/*CDKN1A* transcripts were quantified by qPCR and normalized to *HMBS* mRNA content in control (black bars) and ALMS fibroblasts (white bars). Results are reported as mean values ± SD and are expressed as fold change with respect to controls, arbitrarily set as 1 for each transcript. (b) Telomere lengths estimated by real-time PCR (T/S value) in control (black bars) and ALMS fibroblasts (white bars) are reported as mean values ± SD. Fibroblasts were analyzed at the same passages in culture (XII–XIV); no correlation was found between T/S value, the age of subjects or the number of passages.
(DOC)

Figure S2 **Senescence-associated β-galactosidase activity of control and ALMS fibroblasts.** Control (C1, C2, C3) and ALMS fibroblasts (PT1, PT2, PT3, PT4) at similar passages in culture (XII–XIV) were fixed and stained for β-galactosidase activity at pH 6.0. 293 cells (XXX passage) and 293 cells transiently transfected with plasmid encoding for β-gal were used as positive controls. Magnification: 32×.
(DOC)

Figure S3 **Size and shape of ALMS fibroblasts.** (a) Fibroblasts of healthy controls (C1, C2) and ALMS patients (PT1, PT2, PT4) were grown on standard tissue culture (2D cultures) coverslips and stained with hematoxylin-eosin (magnification 20×). (b) ALMS fibroblasts were compared with controls by flow cytometric analysis and the resulting forward (FSC-H) and side light scatter (SSC-H) mean intensity values and SEM are shown for each subject. The statistical analysis of SSC-H was carried out with the Kolmogorov-Smirnov test which is significant (value >8) for PT1 and PT3 *vs* C1 = 26.5 and also for PT1 and PT3 *vs* C3 = 11.3.
(DOC)

Figure S4 **Motility assay and ultrastructural evaluation of ALMS fibroblasts.** (a) Fibroblasts of healthy controls (C1, C3) and (b) ALMS patients (PT1, PT3) were cultured in HYAFF-11™ scaffolds (3D-cultures) and stained with hematoxylin-eosin (magnification 5×). Transmission electron microscopy was performed in healthy controls (C1, C3) (c–d) and ALMS fibroblasts (PT1, PT3, PT4) (e–f) on 3D-cultured cells. Control fibroblasts showed a normal phenotype, with cytoplasm rich in perpendicular oriented microfilaments (c, rings) and the presence of some pinocytic vesicles (d, arrows). In contrast, in ALMS fibroblasts microfilaments (e, rings) were arranged in a unique direction, parallel to the long axis of the cells. A large number of exocytic vesicles (f, arrows and ring) suggest an active secretion. Magnification: c = 40000×; d(C1) = 25000×; d(C3)30000; e = 30000×; f = 25000.
(DOC)

Figure S5 **Relative mRNA levels of collagens in ALMS fibroblasts.** *COL1A1*, *COL3A1*, *COL4A1*, *COL5A1*, *COL5A2*, *COL8A1*, *COL11A1*, *COL12A1*, *COL15A1* transcripts were quantified by qPCR in ALMS fibroblasts (ALMS) and controls (CONTROLS). Results are reported as threshold cycle (Ct) for each specific qPCR reaction and are expressed as mean values ± SEM.
(DOC)

Figure S6 **Increased mRNA expression of ECM components in ALMS fibroblasts.** (a) *POSTN*, (b) *ACTA2*, (c) *COL1A1*, (d) *COL3A1*, (e) *COL4A1*, (f) *COL5A1*, (g) *COL5A2*, (h) *COL8A1*, (i) *COL11A1*, (l) *COL12A1*, (m) *COL15A1* transcripts were quantified by qPCR and normalized to *HMBS* mRNA content in control (black bars) and ALMS fibroblasts (white bars). Results are reported as mean values ± SEM arbitrary units (AU) ratio. *P<0.05, ** *P*<0.01, *** *P*<0.001 ALMS fibroblasts *versus* controls.
(DOC)

Figure S7 **Pro-fibrotic factors increase** ***COL1A1*** **expression and cellular proliferation in ALMS fibroblasts.** (a) Fibroblasts of healthy controls and ALMS patients were stimulated with TGF-β (10 ng/ml) in 5% FBS SM. *COL1A1* mRNA was quantified by qPCR and normalized to *HMBS* mRNA content. Results are given as fold increase versus unstimulated controls (0 h), set as 1, and presented as mean values ± SEM. **P<0.01 stimulated (48 h) *versus* unstimulated (0 h) ALMS fibroblasts; # *P*<0.05 stimulated ALMS fibroblasts *versus* stimulated control fibroblasts (48 h). (b) The effect of treatment with TGF-β (10 ng/ml), CTGF (100 ng/ml) and FGF2 (10 ng/ml) in 2% FBS SM for 72 hours on cellular proliferation was evaluated in ALMS and control fibroblasts using the CellTiter-Glo® Luminescent Cell Viability Assay (Promega). Results are given as % of viable cells with respect to unstimulated cells, indicated as 100%. ***P<0.001 stimulated with FGF2 *versus* unstimulated ALMS fibroblasts.
(DOC)

Figure S8 **ALMS fibroblasts are resistant to cell death induced by apoptotic stimuli.** Fibroblasts of healthy controls (black bars) and ALMS patients (white bars) were stimulated with (a) thapsigargin (100 nM for 48 hours), (b) C_2_-ceramide (100 µM for 24 hours), (c) cycloheximide (100 µg/ml for 24 hours), (d) staurosporine (100 nM for 48 hours) and TNF-α (100 ng/ml for 48 hours) in 10% FBS SM. The % of viable cells was determined by MTT assay and shown as mean values ± SEM with respect to unstimulated cells indicated as 100%. *P<0.05, ***P<0.001 cell viability of ALMS fibroblasts *versus* controls.
(DOC)

Figure S9 **ALMS fibroblasts are resistant to cell death induced by apoptotic stimuli.** (a) Control (C3) and ALMS (PT2) fibroblasts were treated with THAP (100 nM for 48 hours), stained with PI and analyzed by flow cytometry. Results are presented as histograms of cell cycle phase distribution and the reported % represents the increase in sub-G_1_/G_0_ population (M1 region). (b) Control (C2) and ALMS (PT3) fibroblasts were treated with THAP (100 nM for 48 and 72 hours). Cells were labelled by TUNEL and PI and analyzed by flow cytometry. Each cytogram shows the TUNEL positivity (ordinate) with respect to DNA content (abscissa), upon 48 and 72 hours of THAP-treatment respectively.
(DOC)

Figure S10 **ALMS fibroblasts are resistant to apoptosis.** Control (C2, C3) and ALMS fibroblasts (PT2, PT3, PT4) were grown on glass coverslips and stimulated with CX (100 µg/ml for 24 hours) and THAP (100 nM for 48 hours). Cells were fixed and stained for fluorescence *in situ* DNA end-labelling (TUNEL) (green stain); nuclei were counter-stained with DAPI (blue stain) or with PI (red stain). Magnification = 20×.
(DOC)

Table S1 **Primer sequences and conditions used in qPCR.** *POSTN* = periostin, *ACTA* = actin, alpha-2, smooth muscle, aorta, *COL1A1* = collagen, type I, alpha 1; *COL3A1* = collagen, type III, alpha 1; *COL4A1* = collagen, type IV, alpha 1; *COL5A1* = collagen, type V, alpha 1; *COL5A2* = collagen, type V, alpha 2; *COL8A1* = collagen, type VIII, alpha 1; *COL11A1* = collagen, type XI, alpha 1; *COL12A1* = collagen, type XII, alpha 1; *COL15A1* = collagen, type XV, alpha 1; *HMBS* = hydroxymethylbilane synthase; *CDKN1A = *cyclin-dependent kinase inhibitor 1A (p21, Cip1); *MYC* = v-myc myelocytomatosis viral oncogene homolog (avian); *SHC1* (p66 isoform) = SHC (Src homology 2 domain containing) transforming protein 1; *SIRT1* = sirtuin 1; *SIRT5* = sirtuin 5.
(DOC)

Table S2 **Gene expression modulation in ALMS** ***versus*** **control fibroblasts.** Differentially expressed genes, identified by microarray experiments, were analyzed and grouped by functions. Tables reported only informative genes for each class. The columns show Gene Symbol, Gene Bank, Unigene accession number, gene description and the relative fold change. Each reported gene passed SAM statistic.
(DOC)
