# Supplementary material for: ALMS1-Deficient Fibroblasts Over-Express Extra-Cellular Matrix Components, Display Cell Cycle Delay and Are Resistant to Apoptosis
Source: PLoS One. 2011 Apr 26;6(4):e19081. doi: 10.1371/journal.pone.0019081 (PMC3082548; doi:10.1371/journal.pone.0019081)
Supplement: Table S1 — Primer sequences and conditions used in qPCR. POSTN = periostin, ACTA = actin, alpha-2, smooth muscle, aorta, COL1A1 = collagen, type I, alpha 1; COL3A1 = collagen, type III, alpha 1; COL4A1 = collagen, type IV, alpha 1; COL5A1 = collagen, type V, alpha 1; COL5A2 = collagen, type V, alpha 2; COL8A1 = collagen, type VIII, alpha 1; COL11A1 = collagen, type XI, alpha 1; COL12A1 = collagen, type XII, alpha 1; COL15A1 = collagen, type XV, alpha 1; HMBS = hydroxymethylbilane synthase; CDKN1A = cyclin-dependent kinase inhibitor 1A (p21, Cip1); MYC = v-myc myelocytomatosis viral oncogene homolog (avian); SHC1 (p66 isoform) = SHC (Src homology 2 domain containing) transforming protein 1; SIRT1 = sirtuin 1; SIRT5 = sirtuin 5. (DOC) [file pone.0019081.s011.doc]

| ***GENE*** | **FORWARD** | **REVERSE** | **AMPLICON** | **[PRIMERS]**  **nmol/l** | **CYCLING**  **PARAMETERS** |
| --- | --- | --- | --- | --- | --- |
| ***POSTN*** | 5'-TGG AAA CCA TCG  GAG GCA AA-3' | 5'- ARG TGA ATC GCA  CCG TTT CTC C-3' | 115 bp | F=300  R=300 | 95°C x 15 sec  60°C x 1 min |
| ***ACTA2*** | 5'-AAG GCC AAC CGG  GAG AAA AT-3' | 5'-ATT GTG GGT GAC  ACC ATC TCC A-3' | 150 bp | F=300  R=300 | 95°C x 15 sec  60°C x 1 min |
| ***COL1A1*** | 5'-CCT GGA TGC CAT  CAA AGT CT-3' | 5'-TCT TGT CCT TGG  GGT TCT TG-3' | 116 bp | F=300  R=300 | 95°C x 15 sec  60°C x 1 min |
| ***COL3A1*** | 5'-AAG AAT TTG GTG  TGG ACG TTG-3' | 5'-TTT TGT CGG TCA  CTT GCA CT-3' | 136 bp | F=300  R=300 | 95°C x 15 sec  60°C x 1 min |
| ***COL4A1*** | 5'-CCA GGA TTT CAA  GGT CCA AA-3' | 5'-CTC CCC TTT GAT  GAT GTC GT-3' | 138 bp | F=100  R=100 | 95°C x 15 sec  60°C x 30 sec  72°C x 30 sec |
| ***COL5A1*** | 5'-CCT GAC CCT GGA  CAG TGA AG-3' | 5'-GGC TCC TTC CCT  CTG TTC TC-3' | 110 bp | F=300  R=300 | 95°C x 15 sec  60°C x 1 min |
| ***COL5A2*** | 5'-TCA AAA GAA GCC  TCC CAG AA-3' | 5'-TCT AAG TCA TTT  GCC CCT TTG-3' | 116 bp | F=300  R=300 | 95°C x 15 sec  60°C x 1 min |
| ***COL8A1*** | 5'-ACC ACC CCA GGG  AGA GTA TC-3' | 5'-AAT GCA GGC ATC  TCA TAG GC-3' | 120 bp | F=300  R=300 | 95°C x 15 sec  60°C x 30 sec  78°C x 30 sec |
| ***COL11A1*** | 5'-GCA TTT TGA TGC  TTT ATT CAA GG-3' | 5'-CAC ACA TTT CCC  TGT CCA AA-3' | 123 bp | F=300  R=300 | 95°C x 15 sec  60°C x 1 min |
| ***COL12A1*** | 5'-ACA TGC CGT GTG  CCT TTA GT-3’ | 5'-AAC TGC CCG CTC  GAA ATA C-3’ | 108 bp | F=300  R=300 | 95°C x 15 sec  60°C x 1 min |
| ***COL15A1*** | 5'-AGC AAC CCA CAT  CAG CTT CT-3' | 5'-ATG CTC GGT AGG  TGG ACA AC-3' | 172 bp | F=300  R=300 | 95°C x 15 sec  60°C x 1 min |
| ***HMBS*** | 5’-GGC AAT GCG GCT  GCA A-3’ | 5’-GGG TAC CCA CGC  GAA TCA C-3’ | 60 bp | F=300  R=300 | 95°C x 15 sec  60°C x 1 min |
| ***CDKN1A*** | 5’- TGG AGA CTC TCA  GGG TCG AAA A-3’ | 5’- GGC TTC CTC TTG  GAG AAG ATC A-3’ | 90 bp | F=300  R=300 | 95°C x 15 sec  60°C x 1 min |
| ***MYC*** | 5’- TTT TTC GGG TAG  TGG AAA ACC A-3’ | 5’- TCC TCC TCG TCG  CAG TAG AAA T-3’ | 124 bp | F=300  R=300 | 95°C x 15 sec  60°C x 1 min |
| ***SHC1***  ***(p66 isoform)*** | 5’- TCA ATG GGG ACT  TCC TGG TA-3’ | 5’- TAG TCC GAA CCA  CAC CCT CA-3’ | 123 bp | F=300  R=300 | 95°C x 15 sec  60°C x 1 min |
| ***SIRT1*** | 5’- GAG TGG CAA AGG  AGC AGA-3’ | 5’- TCT GGC ATG TCC  CAC TAT C-3’ | 165 bp | F=300  R=300 | 95°C x 15 sec  60°C x 1 min |
| ***SIRT5*** | 5’- TCG TGG TCA TCA  CCC AGA ACA T-3’ | 5’- TCT CAG CCA CAA  CTC CAC AAG A-3’ | 120 bp | F=300  R=300 | 95°C x 15 sec  60°C x 1 min |
