# Supplementary material for: ALMS1-Deficient Fibroblasts Over-Express Extra-Cellular Matrix Components, Display Cell Cycle Delay and Are Resistant to Apoptosis
Source: PLoS One. 2011 Apr 26;6(4):e19081. doi: 10.1371/journal.pone.0019081 (PMC3082548; doi:10.1371/journal.pone.0019081)
Supplement: Table S2 — Gene expression modulation in ALMS versus control fibroblasts. Differentially expressed genes, identified by microarray experiments, were analyzed and grouped by functions. Tables reported only informative genes for each class. The columns show Gene Symbol, Gene Bank, Unigene accession number, gene description and the relative fold change. Each reported gene passed SAM statistic. (DOC) [file pone.0019081.s012.doc]

**CELL CYCLE RELATED GENES**

| **GENE SYMBOL** | **GENE BANK** | **UNIGENE** | **DESCRIPTION** | **FOLD CHANGE**  **(ALMS *vs* CONTROL)** |
| --- | --- | --- | --- | --- |
| cell cycle progression associated genes | | | | |
| IL7R  CKS2  CCNE2  PBK  E2F7  CDC2  DTL  TMPO  UBE2C  CCNA2  CDC6  CCNB1  NCAPG2 | NM_002185  NM_001827  NM_057749  NM_018492  BC016658  NM_001786  NM_016448  NM_003276  NM_007019  NM_001237  NM_001254  NM_031966  NM_017760 | 237868  83758  30464  104741  144687  334562  126774  11355  93002  85137  69563  23960  18616 | Interleukin 7 receptor  CDC28 protein kinase 2  Cyclin E2  T-LAK cell-originated protein kinase  Homo sapiens, Similar to growth accentuating protein 43, mRNA  Cell division cycle 2, G1 to Sand G2 to M  Denticleless homolog (Drosophila)  Thymopoietin  Ubiquitin-conjugating enzyme E2C  Cyclin A2  CDC6 cell division cycle6 homolog (S.cerevisiae)  Cyclin B1  non-SMC condensin II complex, subunit G2 | -0.98  -0.94  -0.92  -0.91  -0.9  -0.86  -0.77  -0.73  -0.7  -0.67  -0.62  -0.56  -0.52 |
| replication associated genes | | | | |
| RRM2  HELLS  GINS2  RFC4  PRIM2A  PRIM1  RFC3  DTYMK  MCM2  TOP1  MCM5  MCM3  MCM4  MCM6  RPA2  RRM1  TIMELESS  RPA3  TOP2A  RFC5 | NM_001034  AF155827  NM_016095  NM_002916  NM_000947  NM_000946  NM_002915  NM_012145  NM_004526  NM_003286  NM_006739  NM_002388  X74794  NM_005915  NM_002946  NM_001033  NM_003920  NM_002947  NM_001067  NM_007370 | 75319  203963  108196  35120  74519  82741  115474  79006  57101  317  77171  179565  154443  155462  79411  2934  118631  1608  156346  171075 | Ribonucleotide reductase M2 polypeptide  Helicase, lymphoid-specific  GINS complex subunit 2 (Psf2 homolog)  Replication factor C (activator1) 4 (37kD)  Primase, polypeptide 2A (58kD)  Primase, polypeptide 1 (49kD)  Replication factor C (activator1) 3 (38kD)  Deoxythymidylate kinase (thymidylatekinase)  MCM2 mini chromosome maintenance deficient 2, mitotin(S.cerevisiae)  Topoisomerase (DNA)I  MCM5 mini chromosome maintenance deficient 3 (S.cerevisiae)  MCM3 mini chromosome maintenance deficient 3 (S.cerevisiae)  MCM4 mini chromosome maintenance deficient 4 (S.cerevisiae)  MCM6 mini chromosome maintenance deficient 6  Replication protein A2 (32kD)  Ribonucleotide reductase M1 polypeptide  Timeless homolog (Drosophila)  Replication protein A3 (14kD)  Topoisomerase (DNA) II alpha (170kD)  Replication factor C (activator1) 5 (36.5kD) | -1.42  -1.08  -0.96  -0.89  -0.81  -0.77  -0.75  -0.71  -0.68  -0.57  -0.57  -0.56  -0.51  -0.5  -0.48  -0.47  -0.47  -0.45  -0.43  -0.32 |
| centrosome-kinetocore associated genes | | | | |
| SPBC25  AURKA  MAD2L1  RCC1  CDCA8  STMN1  OIP5  BUB1  FAM83D  BLM  CEP55  CENPM  TMPO  H2AFZ  BUB1B  ECT2  CDC20  TPX2  ANLN  CENPF  KIF2C  LMNB2  KIF23  KIF20A  CDC25B  TUBG1 | NM_020675  NM_003158  NM_002358  NM_001269  NM_018101  NM_005563  BC015050  NM_004336  AK055793  NM_000057  NM_018131  NM_024053  NM_003276  NM_002106  NM_001211  NM_018098  NM_001255  NM_012112  NM_018685  NM_016343  NM_006845  M94362  NM_004856  NM_005733  NM_021874  NM_001070 | 21137  333116  79078  84746  48855  250811  116206  98658  70704  36820  14559  208912  11355  119192  36708  122579  82906  9329  62180  77204  69360  334709  270845  73625  153752  21635 | SPC25, NDC80 kinetochore complex component, homolog (S. cerevisiae)  Aurora kinase A  MAD2 mitotic arrest deficient-like 1 (yeast)  Regulator of chromosome condensation 1  Cell division cycle associated 8  Stathmin1/oncoprotein 18  Opa-interacting protein 5  BUB1 budding uninhibited bybenzimidazoles 1 homolog (yeast)  FAM83D family with sequence similarity 83, member D  Bloom syndrome, RecQ helicase-like  Centrosomal protein 55kDa  Centromere protein M  Thymopoietin  H2A histone family, member Z  BUB1 budding uninhibited bybenzimidazoles 1 homolog beta(yeast)  Epithelial cell transforming sequence 2 oncogene  CDC20 cell division cycle 20 homolog (S.cerevisiae)  TPX2, microtubule-associated, homolog (Xenopus laevis)  Anillin, actin binding protein (scraps homolog, Drosophila)  Centromere protein F (350/400kD, mitosin)  Kinesin-like 6 (mitotic centromere-associated kinesin)  Lamin B2  Kinesin-like 5(mitotic kinesin-like protein 1)  RAB6 interacting, kinesin-like (rabkinesin6)  Cell division cycle 25B  Tubulin, gamma 1 | -0.98  -0.89  -0.88  -0.86  -0.85  -0.84  -0.83  -0.75  -0.74  -0.74  -0.73  -0.73  -0.73  -0.72  -0.72  -0.71  -0.63  -0.62  -0.61  -0.6  -0.52  -0.51  -0.5  -0.46  -0.46  -0.34 |

**EXTRACELLULAR MATRIX/FIBROSIS RELATED GENES**

| **GENE**  **SYMBOL** | **GENE BANK** | **UNIGENE** | **DESCRIPTION** | **FOLD CHANGE (ALMS *vs* CONTROL)** |
| --- | --- | --- | --- | --- |
| HAPLN1  ASPN  PRELP  COL15A1  COL8A1  COL11A1  FGFR2  ITGA11  SGCD  IGFBP5  DPT  ADAM12  COL5A2  COL5A1  COL3A1  COL1A1  HAS2  COL12A1  COL4A1  P4HA1  CTGF  ACTA2  SDC2  IGFBP3  LEPRE1  LUM  MFAP4  MFAP2  SERPINH1  CYR61  SPARC  NOV  CD36  CCR2  IL7  LEPREL1 | NM_001884  NM_017680  NM_002725  NM_001855  AL359062  NM_001854  NM_023028  AF109681  NM_000337  NM_000599  NM_001937  NM_003474  NM_000393  NM_000093  NM_000090  NM_000088  NM_005328  NM_004370  NM_001845  NM_000917  NM_001901  NM_001613  J04621  M35878  NM_022356  NM_002345  L38486  NM_017459  NM_001235  NM_001554  NM_003118  NM_002514  NM_000072  NM_000647  NM_000880  NM_018192 | 2799  10760  76494  83164  41271  82772  278581  256297  151899  3488  80552  8850  82985  146428  119571  172928  159226  101302  119129  76768  75511  195851  1501  77326  10114  79914  296049  83551  9930  8867  111779  235935  75613  395  72927  42824 | Cartilage linking protein 1  Asporin  Proline arginine-rich end leucine-rich repeat protein  Collagen, type XV, alpha 1  Homo sapiens mRNA full length insert cDNA clone EUROIMAGE1913076  Collagen, typeXI, alpha 1  Fibroblast growth factor receptor 2  Integrin,alpha11  Sarcoglycan, delta (35kDa dystrophin-associated glycoprotein)  IGFBP5 insulin-like growth factor binding protein 5  Dermatopontin  A disintegrin and metalloproteinase domain 12 (meltrin alpha)  Collagen, typeV, alpha 2  Collagen, typeV, alpha 1  Collagen, typeIII, alpha 1  Collagen, typeI, alpha 1  Hyaluronan synthase 2  Collagen, typeXII, alpha 1  Collagen, typeIV, alpha 1  Procollagen-proline, 2-oxoglutarate4-dioxygenase  Connective tissue growth factor  Actin, alpha 2, smooth muscle, aorta  Syndecan 2  Insulin-like growth factor binding protein 3  Growth suppressor 1  Lumican  Microfibrillar-associated protein 4  Microfibrillar-associated protein 2  Serine (or cysteine) proteinase inhibitor, (heat shock protein 47), member2  Cysteine-rich, angiogenic inducer, 61  Secreted protein, acidic, cysteine-rich (osteonectin)  Nephroblastoma overexpressed gene  CD36 molecule (thrombospondin receptor)  Chemokine(C-Cmotif) receptor2  Interleukin7  Leprecan-like 1 | 3.35  3.15  2.81  2.45  2.13  2.07  1.76  1.5  1.43  1.42  1.38  1.36  1.34  1.1  1.08  1.08  1.04  1.01  1  0.99  0.95  0.82  0.81  0.8  0.77  0.74  0.71  0.68  0.51  0.51  0.49  -1.34  -1.25  -0.92  -0.87  -0.65 |

A**DHESION/MOTILITY RELATED GENES**

| **GENE**  **SYMBOL** | **GENE BANK** | **UNIGENE** | **DESCRIPTION** | **FOLD CHANGE**  **(ALMS *vs* CONTROL)** |
| --- | --- | --- | --- | --- |
| POSTN  WISP1  IGFBP5  DPT  CSPG2  ITGB5  SCD2  TNS1  LUM  LGALS3BP  PTK7  MFAP4  MFAP2  LIMS2  AMIGO2  EDG2  CD9  HMMR  EPDR1  TYRO3  GJA7 | NM_006475  AK027294  NM_000599  NM_001937  U16306  NM_002213  J04621  AK057328  NM_002345  NM_005567  NM_002821  L38486  NM_017459  NM_017980  AC004010  NM_057159  NM_001769  NM_012484  AY027862  NM_006293  NM_005497 | 136348  9812  3488  80552  81800  149846  1501  9973  79914  79339  90572  296049  83551  127273  121520  75794  1244  72550  46721  301  43761 | Osteoblast specific factor 2 (fasciclin I-like)  WNT1 inducible signaling pathway protein 1  IGFBP5 insulin-like growth factor binding protein 5  Dermatopontin  Chondroitin sulfate proteoglycan 2 (versican)  Integrin, beta 5  Syndecan 2  Tensin 1  Lumican  Lectin, galactoside-binding, soluble,3 binding protein  PTK7 protein tyrosine kinase 7  Microfibrillar-associated protein 4  Microfibrillar-associated protein 2  LIM and senescent cell antigen-like domains 2  Adhesion molecule with Ig-like domain 2  Endothelial differentiation, lysophosphatidicacid G-protein-coupled receptor,2  CD9 antigen(p24)  Hyaluronan-mediated motility receptor (RHAMM)  Ependymin related protein 1 (zebrafish)  TYRO3 protein tyrosine kinase  Gap junction protein, alpha7, 45kD(connexin45) | 4.12  2.33  1.42  1.38  1.35  0.88  0.81  0.75  0.74  0.72  0.71  0.71  0.68  0.67  -1.11  -1.08  -0.86  -0.65  -0.6  -0.56  -0.49 |

**APOPTOSIS RELATED GENES**

| **GENE SYMBOL** | **GENE BANK** | **UNIGENE** | **DESCRIPTION** | **FOLD CHANGE (ALMS *vs* CONTROL)** |
| --- | --- | --- | --- | --- |
| POSTN  WISP1  EDIL3  MEF2C  ENPP2  IGF1R  GAS6  FOS  CYR61  IL24  GULP1  TFPI2  NALP1  MAF  SALL2  CUGBP2  PCNA  AKAP8 | NM_006475  AK027294  NM_005711  L08895  NM_006209  NM_000875  NM_000820  NM_005252  NM_001554  NM_006850  NM_016315  NM_006528  NM_021730  AF055376  X98834  AF090693  NM_002592  NM_005858 | 136348  9812  129764  78995  174185  104679  78501  25647  8867  315463  107056  295944  7212  30250  79971  211610  78996  25059 | Osteoblast specific factor 2 (fasciclin I-like)  WNT1 inducible signaling pathway protein 1  EGF-like repeat sand discoidin I-like domains 3  MADS box transcription enhancer factor 2, polypeptide C (myocyte enhancer factor 2C)  Ecto nucleotide pyrophosphatase/ phosphor diesterase 2 (autotaxin)  Insulin-like growth factor 1 receptor  Growth arrest-specific 6  V-fos FBJ murine osteosarcoma viral oncogene homolog  Cysteine-rich, angiogenic inducer, 61  Interleukin 24  GULP, engulfment adaptor PTB domain containing 1  Tissue factor pathway inhibitor 2  Hypothetical protein PP1044  V-maf musculo aponeurotic fibrosarcoma oncogene homolog (avian)  Sal-like2 (Drosophila)  CUG triplet repeat, RNA binding protein 2  Proliferating cell nuclear antigen  A kinase (PRKA) anchor protein 8 | 4.12  2.33  2.15  1.5  0.98  0.79  0.76  0.65  0.51  -1.72  -1.4  -0.99  -0.94  -0.92  -0.74  -0.69  -0.45  -0.34 |
