# Supplementary material for: ALMS1-Deficient Fibroblasts Over-Express Extra-Cellular Matrix Components, Display Cell Cycle Delay and Are Resistant to Apoptosis
Source: PLoS One. 2011 Apr 26;6(4):e19081. doi: 10.1371/journal.pone.0019081 (PMC3082548; doi:10.1371/journal.pone.0019081)
Supplement: Supplementary file 3 [file pone.0019081.s001.doc]

a

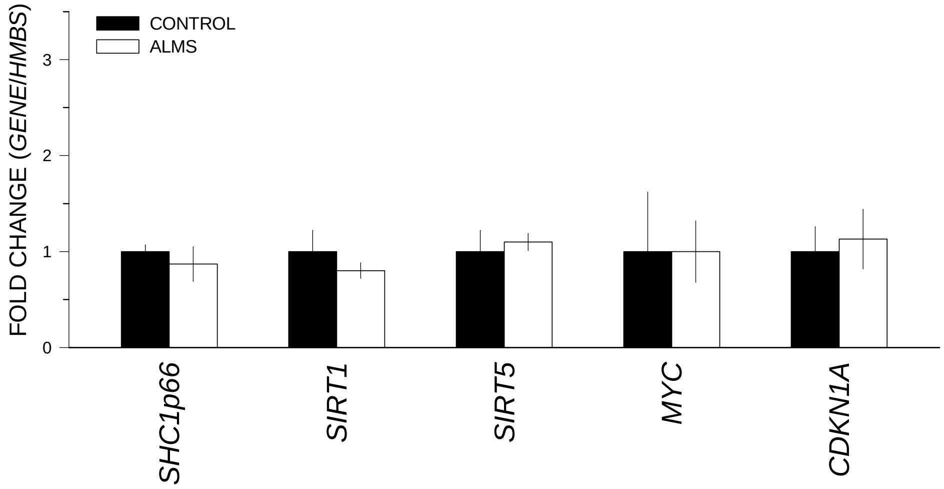

CONTROL
ALMS
FOLD CHANGE (GENE/HMBS)
0
1
2
3
SHC1p66
SIRT1
SIRT5
MYC
CDKN1A

b

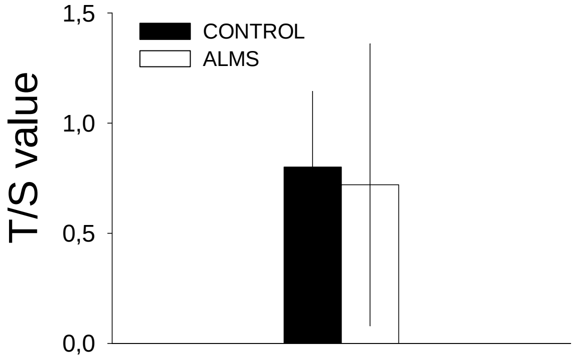

CONTROL
ALMS
T/S value
0,0
0,5
1,0
1,5
