# Supplementary material for: ALMS1-Deficient Fibroblasts Over-Express Extra-Cellular Matrix Components, Display Cell Cycle Delay and Are Resistant to Apoptosis
Source: PLoS One. 2011 Apr 26;6(4):e19081. doi: 10.1371/journal.pone.0019081 (PMC3082548; doi:10.1371/journal.pone.0019081)

PT2

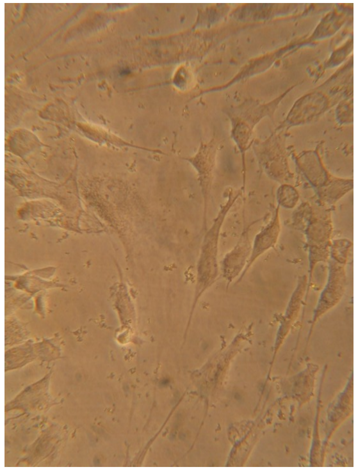

PT1

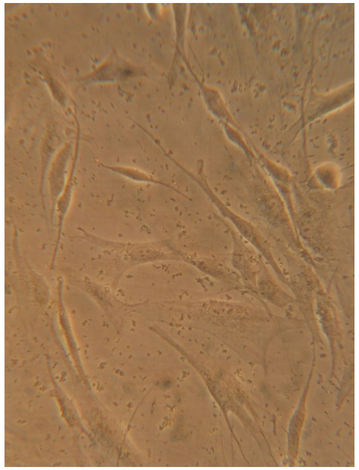

PT4

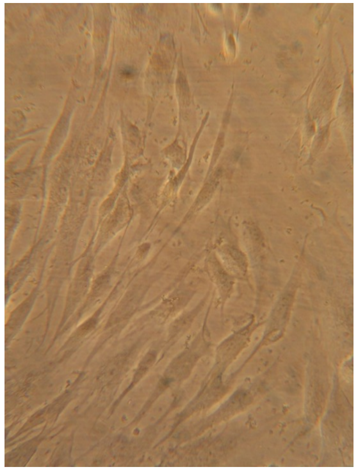

PT3

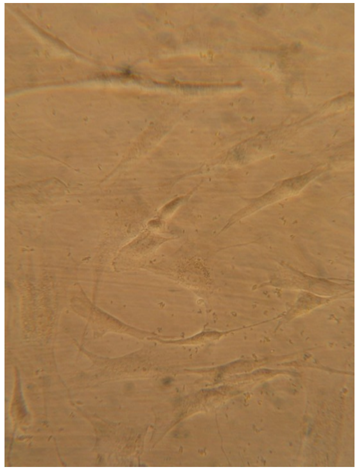

293

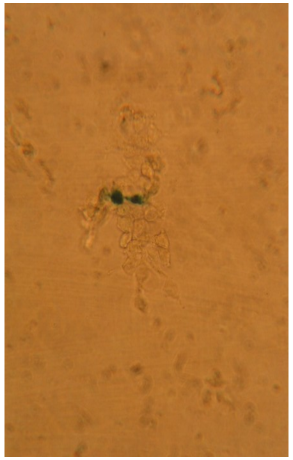

293βgal

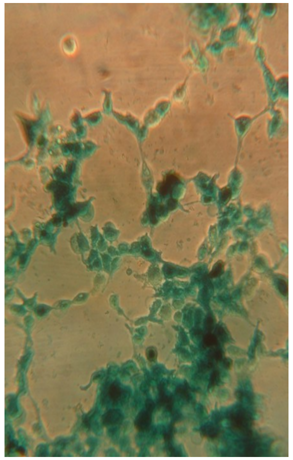

C3

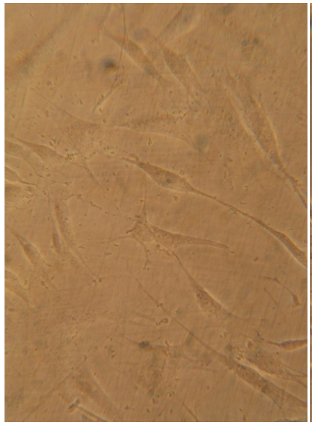

C2

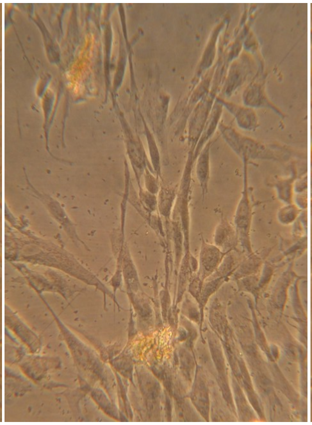

C1

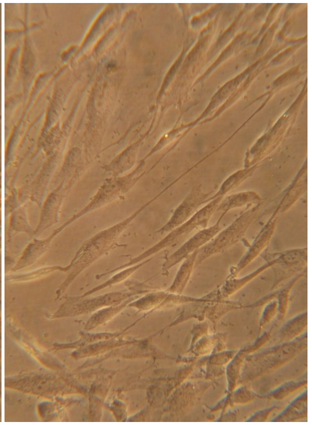

Supplement: Supplementary file 4 [file pone.0019081.s002.doc]
