# Supplementary material for: ALMS1-Deficient Fibroblasts Over-Express Extra-Cellular Matrix Components, Display Cell Cycle Delay and Are Resistant to Apoptosis
Source: PLoS One. 2011 Apr 26;6(4):e19081. doi: 10.1371/journal.pone.0019081 (PMC3082548; doi:10.1371/journal.pone.0019081)
Supplement: Supplementary file 5 [file pone.0019081.s003.doc]

a

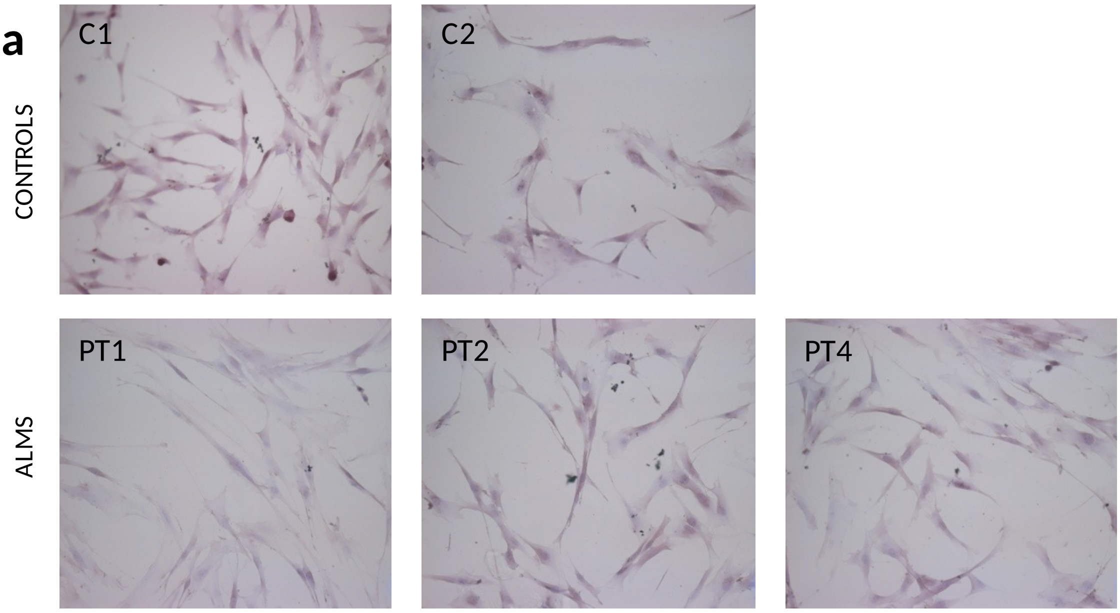

b

	FSC-H		SSC-H	
	MEAN	SEM	MEAN	SEM
C1	467.345	3.372899	183.555	4.391133
C2	466.965	7.516545	192.015	0.784889
C3	460.58	4.327494	220.555	7.078139
PT1	430.17	10.13991	233.505	12.91884
PT2	446.595	2.071823	179.48	0.735391
PT3	477.32	13.15219	232.575	10.44397
PT4	477.275	11.91475	182.175	9.46816
