# Supplementary material for: ALMS1-Deficient Fibroblasts Over-Express Extra-Cellular Matrix Components, Display Cell Cycle Delay and Are Resistant to Apoptosis
Source: PLoS One. 2011 Apr 26;6(4):e19081. doi: 10.1371/journal.pone.0019081 (PMC3082548; doi:10.1371/journal.pone.0019081)
Supplement: Supplementary file 6 [file pone.0019081.s004.doc]

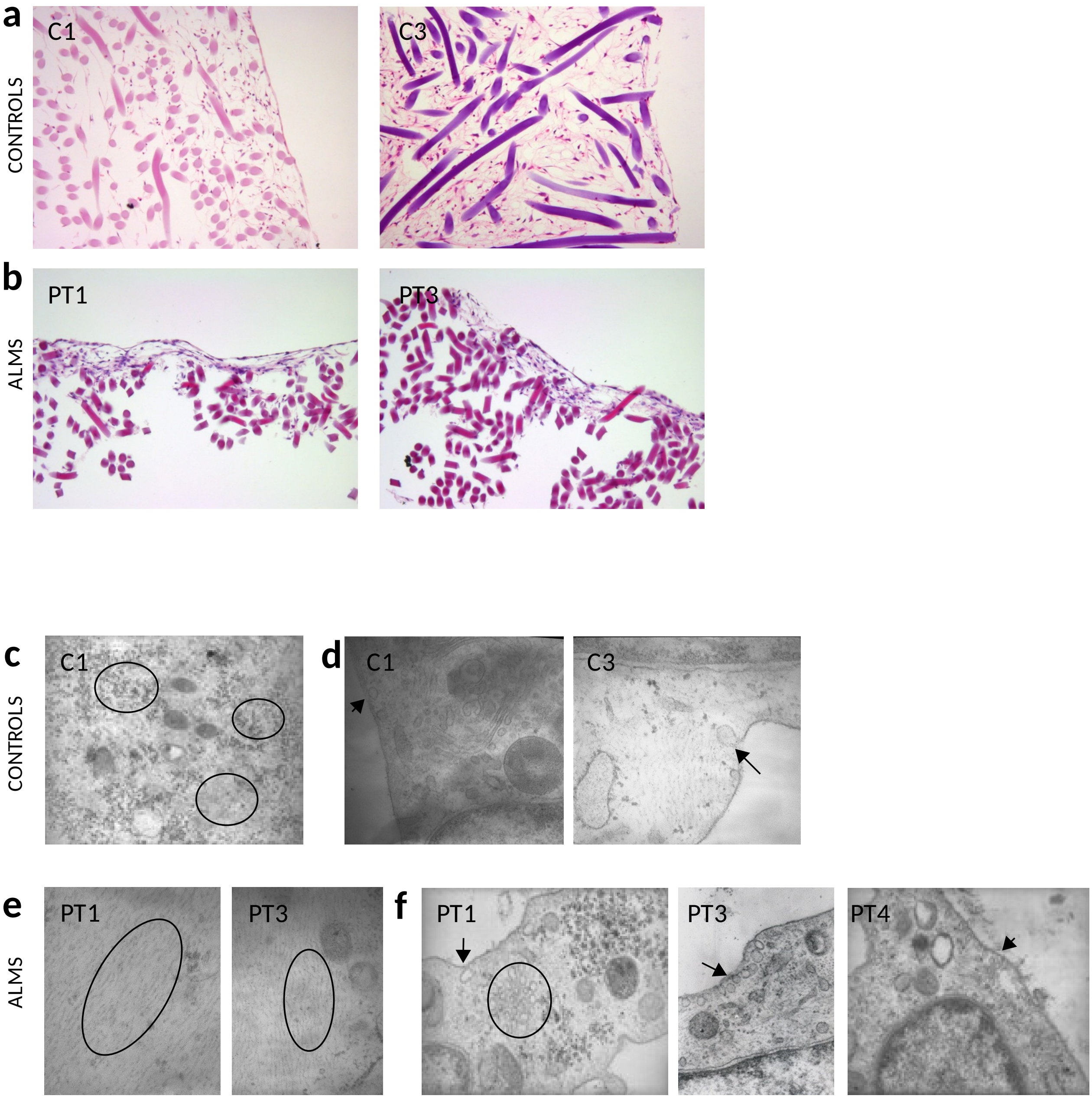

a
CONTROLS
C1
C3
b
ALMS
PT1
PT3
c
CONTROLS
C1
d
C1
C3
e
ALMS
PT1
PT3
f
PT1
PT3
PT4
