# Supplementary material for: ALMS1-Deficient Fibroblasts Over-Express Extra-Cellular Matrix Components, Display Cell Cycle Delay and Are Resistant to Apoptosis
Source: PLoS One. 2011 Apr 26;6(4):e19081. doi: 10.1371/journal.pone.0019081 (PMC3082548; doi:10.1371/journal.pone.0019081)
Supplement: Supplementary file 7 [file pone.0019081.s005.doc]

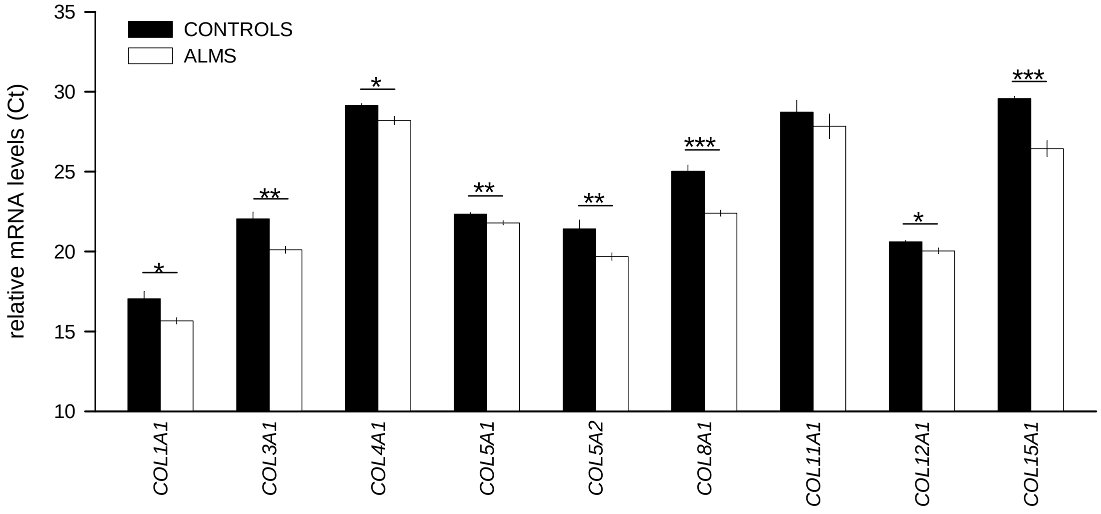

35
30
25
20
15
10
relative mRNA levels (Ct)
CONTROLS
ALMS
*
**
*
**
**
***
*
***
COL1A1
COL3A1
COL4A1
COL5A1
COL5A2
COL8A1
COL11A1
COL12A1
COL15A1
