# Supplementary material for: ALMS1-Deficient Fibroblasts Over-Express Extra-Cellular Matrix Components, Display Cell Cycle Delay and Are Resistant to Apoptosis
Source: PLoS One. 2011 Apr 26;6(4):e19081. doi: 10.1371/journal.pone.0019081 (PMC3082548; doi:10.1371/journal.pone.0019081)
Supplement: Supplementary file 8 [file pone.0019081.s006.doc]

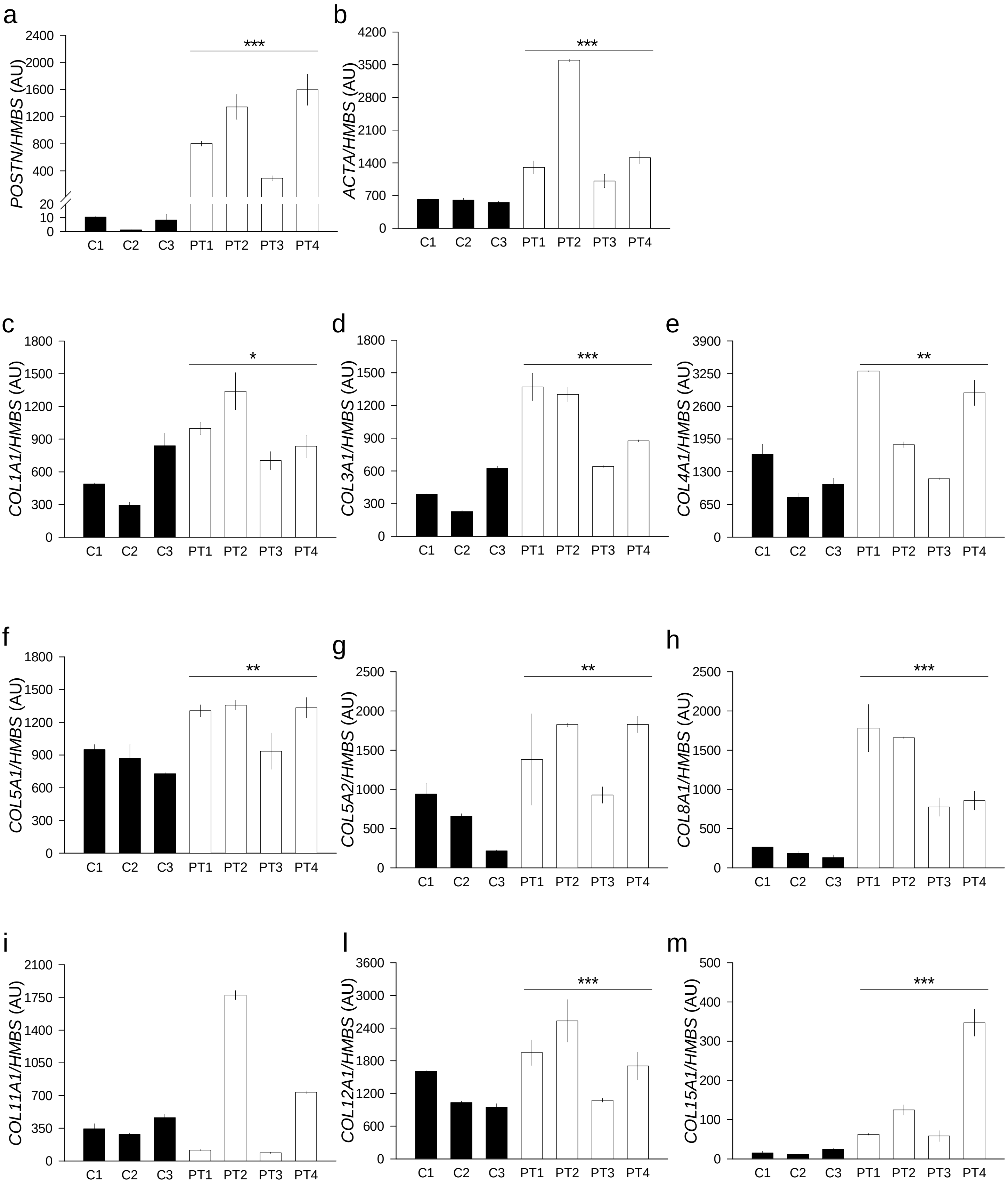
a
POSTN/HMBS (AU)
2400
2000
1600
1200
800
400
20
10
0
***
C1 C2 C3 PT1 PT2 PT3 PT4
b
ACTA/HMBS (AU)
4200
3500
2800
2100
1400
700
0
***
C1 C2 C3 PT1 PT2 PT3 PT4
c
COL1A1/HMBS (AU)
1800
1500
1200
900
600
300
0
*
C1 C2 C3 PT1 PT2 PT3 PT4
d
COL3A1/HMBS (AU)
1800
1500
1200
900
600
300
0
***
C1 C2 C3 PT1 PT2 PT3 PT4
e
COL4A1/HMBS (AU)
3900
3250
2600
1950
1300
650
0
**
C1 C2 C3 PT1 PT2 PT3 PT4
f
COL5A1/HMBS (AU)
1800
1500
1200
900
600
300
0
**
C1 C2 C3 PT1 PT2 PT3 PT4
g
COL5A2/HMBS (AU)
2500
2000
1500
1000
500
0
**
C1 C2 C3 PT1 PT2 PT3 PT4
h
COL8A1/HMBS (AU)
2500
2000
1500
1000
500
0
***
C1 C2 C3 PT1 PT2 PT3 PT4
i
COL11A1/HMBS (AU)
2100
1750
1400
1050
700
350
0
C1 C2 C3 PT1 PT2 PT3 PT4
l
COL12A1/HMBS (AU)
3600
3000
2400
1800
1200
600
0
***
C1 C2 C3 PT1 PT2 PT3 PT4
m
COL15A1/HMBS (AU)
500
400
300
200
100
0
***
C1 C2 C3 PT1 PT2 PT3 PT4
