# Supplementary material for: ALMS1-Deficient Fibroblasts Over-Express Extra-Cellular Matrix Components, Display Cell Cycle Delay and Are Resistant to Apoptosis
Source: PLoS One. 2011 Apr 26;6(4):e19081. doi: 10.1371/journal.pone.0019081 (PMC3082548; doi:10.1371/journal.pone.0019081)
Supplement: Supplementary file 9 [file pone.0019081.s007.doc]

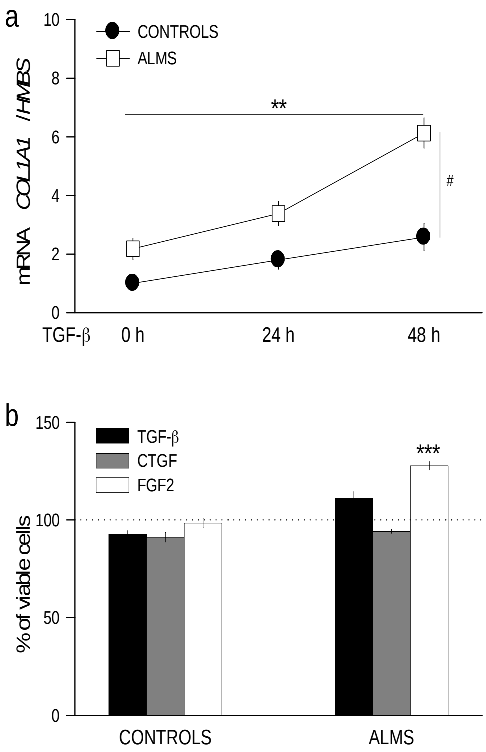

a
CONTROLS
ALMS
**
#
mRNA COL1A1 / HMBS
10
8
6
4
2
0
TGF-β
0 h
24 h
48 h
b
TGF-β
CTGF
FGF2
***
% of viable cells
150
100
50
0
CONTROLS
ALMS
