# Supplementary material for: ALMS1-Deficient Fibroblasts Over-Express Extra-Cellular Matrix Components, Display Cell Cycle Delay and Are Resistant to Apoptosis
Source: PLoS One. 2011 Apr 26;6(4):e19081. doi: 10.1371/journal.pone.0019081 (PMC3082548; doi:10.1371/journal.pone.0019081)
Supplement: Supplementary file 10 [file pone.0019081.s008.doc]

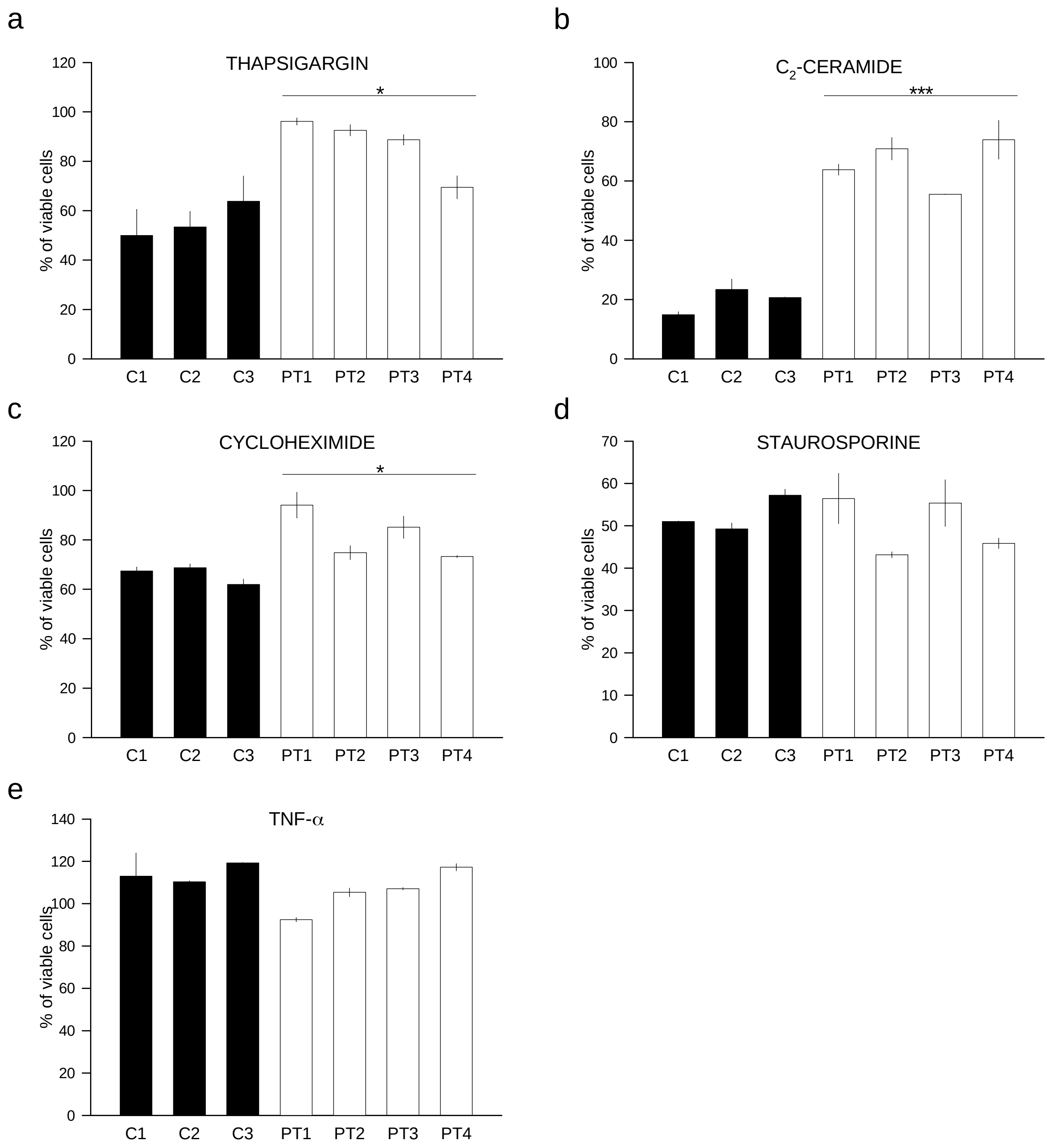
a
THAPSIGARGIN
% of viable cells
120
100
80
60
40
20
0
*
C1 C2 C3 PT1 PT2 PT3 PT4
b
C_2-CERAMIDE
% of viable cells
100
80
60
40
20
0
***
C1 C2 C3 PT1 PT2 PT3 PT4
c
CYCLOHEXIMIDE
% of viable cells
120
100
80
60
40
20
0
*
C1 C2 C3 PT1 PT2 PT3 PT4
d
STAUROSPORINE
% of viable cells
70
60
50
40
30
20
10
0
C1 C2 C3 PT1 PT2 PT3 PT4
e
TNF-α
% of viable cells
140
120
100
80
60
40
20
0
C1 C2 C3 PT1 PT2 PT3 PT4
