# Supplementary material for: ALMS1-Deficient Fibroblasts Over-Express Extra-Cellular Matrix Components, Display Cell Cycle Delay and Are Resistant to Apoptosis
Source: PLoS One. 2011 Apr 26;6(4):e19081. doi: 10.1371/journal.pone.0019081 (PMC3082548; doi:10.1371/journal.pone.0019081)
Supplement: Supplementary file 11 [file pone.0019081.s009.doc]

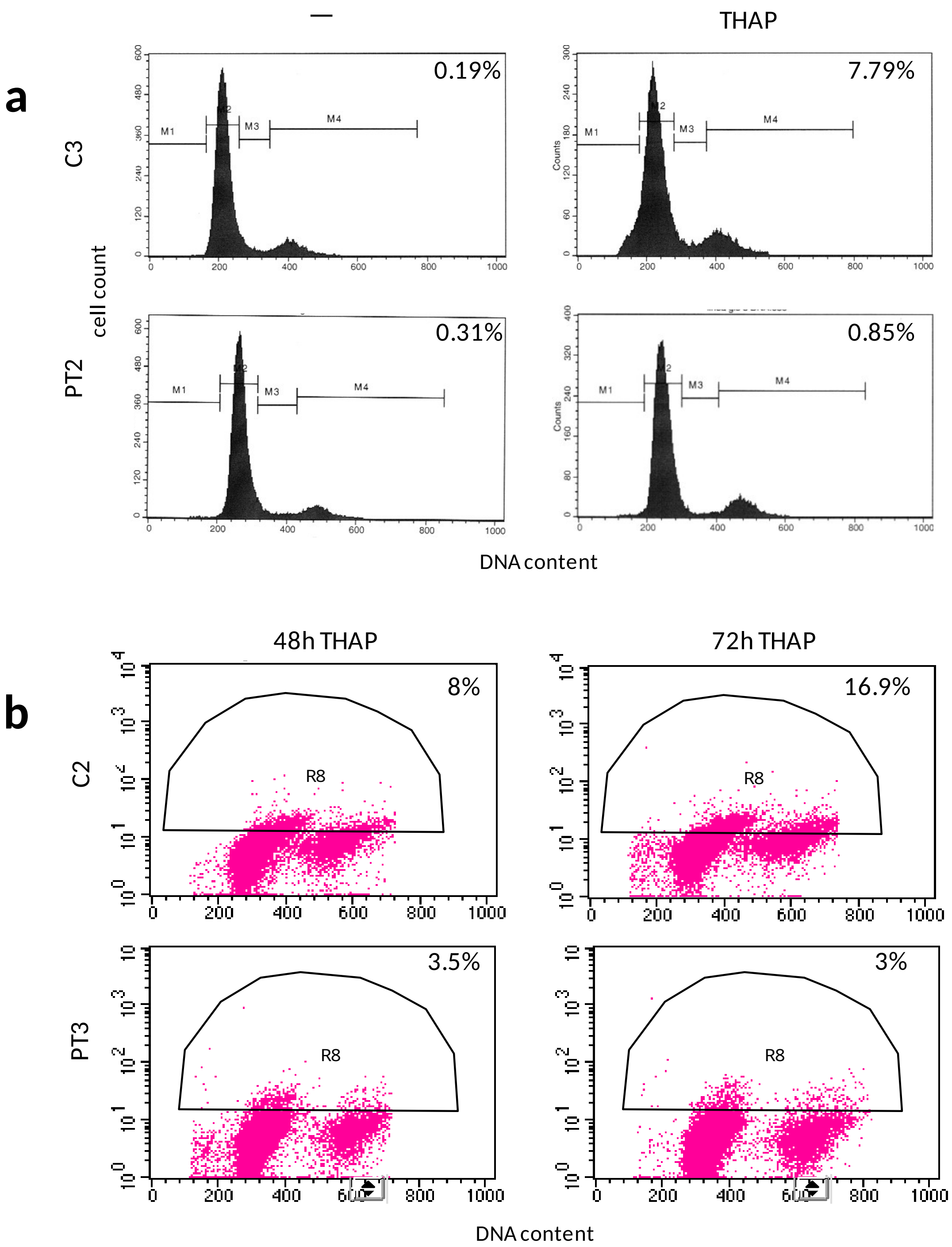
a
—
THAP
C3
PT2
cell count
0.19%
7.79%
0.31%
0.85%
M1
M3
M4
Counts
DNA content
b
48h THAP
72h THAP
C2
PT3
8%
16.9%
3.5%
3%
R8
DNA content
